# Photoswitching fingerprint analysis bypasses the 10-nm resolution barrier

**DOI:** 10.1038/s41592-022-01548-6

**Published:** 2022-08-01

**Authors:** Dominic A. Helmerich, Gerti Beliu, Danush Taban, Mara Meub, Marcel Streit, Alexander Kuhlemann, Sören Doose, Markus Sauer

**Affiliations:** 1 Department of Biotechnology and Biophysics, Biocenter, University of Würzburg, Am Hubland, Würzburg, Germany; 2 Rudolf Virchow Center, Research Center for Integrative and Translational Bioimaging, University of Würzburg, Würzburg, Germany

**Keywords:** Single-molecule biophysics, Nanoscale biophysics

## Abstract

Advances in super-resolution microscopy have demonstrated single-molecule localization precisions of a few nanometers. However, translation of such high localization precisions into sub-10-nm spatial resolution in biological samples remains challenging. Here we show that resonance energy transfer between fluorophores separated by less than 10 nm results in accelerated fluorescence blinking and consequently lower localization probabilities impeding sub-10-nm fluorescence imaging. We demonstrate that time-resolved fluorescence detection in combination with photoswitching fingerprint analysis can be used to determine the number and distance even of spatially unresolvable fluorophores in the sub-10-nm range. In combination with genetic code expansion with unnatural amino acids and bioorthogonal click labeling with small fluorophores, photoswitching fingerprint analysis can be used advantageously to reveal information about the number of fluorophores present and their distances in the sub-10-nm range in cells.

## Abstract (web summary)

Energy transfer between fluorophores is shown to impede SMLM at sub-10-nm spatial resolution. Time-resolved detection and photoswitching fingerprinting analysis are used to determine the number and separation of closely spaced fluorophores.

## Main

Over the past decade, super-resolution fluorescence imaging by single-molecule localization has evolved as a powerful method for subdiffraction-resolution fluorescence imaging of cells and structural investigations of subcellular structures^1,2^. However, although single-molecule localization microscopy (SMLM) methods can now provide a spatial resolution of roughly 20 nm, that is, well below the diffraction limit of light microscopy, they do not provide true molecular resolution of a few nanometers, which is required to comprehensively understand the composition and 3D organization of multiprotein complexes or protein-dense networks in real biological samples such as cells or tissues.

Three central parameters that determine image resolution in SMLM experiments are the localization precision (statistical spread of the measured position coordinates), the localization accuracy (systematic deviation between the measured and true position) and the labeling density. Much attention has focused mainly on improving the localization precision as one of the key determinants of image resolution^3–5^. For instance, the use of sequential structured illumination in combination with single-molecule detection as used in MINFLUX, ROSE, SIMPLE and SIMFLUX allowed to improve the localization precision of direct stochastic optical reconstruction microscopy (dSTORM)^6,7^ using the red-absorbing cyanine dyes Alexa Fluor 647 (AF647) and Cy5 in photoswitching buffer to the 1–5 nm range^8–12^. Such high localization precisions permitted to resolve fluorophores separated by only 6 nm in DNA origami and roughly 10 nm in nuclear pore complexes, respectively^8,12^. However, the results also sparked a debate about the spatial resolution claimed and the reliability of the method^13^. In particular, the images revealed a low detection probability of fluorophores when separated by only a few nanometers evidenced by a high number of incomplete DNA origami and missing protein localizations in the biological samples. On the other hand, these reports demonstrated that anisotropic photon emission of fluorophores due to limited rotational mobility, which has been assumed to cause substantial localization bias^14,15^, can be neglected for highly water-soluble cyanine dyes such as AF647 and Cy5. Hence, the observed low localization probability of fluorophores separated by <10 nm represents a conundrum.

Since site-specific and quantitative labeling of DNA origami with fluorophores is feasible even for sub-10-nm interfluorophore distances, it remains obscured why nanometer localization precisions cannot be translated into molecular resolution with higher reliability. Or, in other words, why does the localization probability severely decrease for interfluorophore distances of <10 nm and how can we bypass this hitherto nonperceived limit? So far, a model that convincingly explains the observed behavior does not exist. To decipher the limits that SMLM methods are facing in the sub-10-nm regime and that cause the observed deterioration in localization probability, we studied DNA origami with different interfluorophore distances. Our data demonstrate that the on/off photoswitching kinetics strongly depends on interfluorophore distance in the sub-10-nm range. We show how photoswitching fingerprint analysis in combination with time-resolved fluorescence detection can overcome these limitations. We demonstrate the concept using DNA origami carrying four fluorophores at different distances and exemplify its translation to biological systems by investigating the stoichiometry and interfluorophore distance of subunits of oligomeric receptors in cells labeled by genetic code expansion (GCE) with unnatural amino acids and click labeling using tetrazine dyes.

## Results

### The 10-nm resolution barrier

**Fig. 1:**
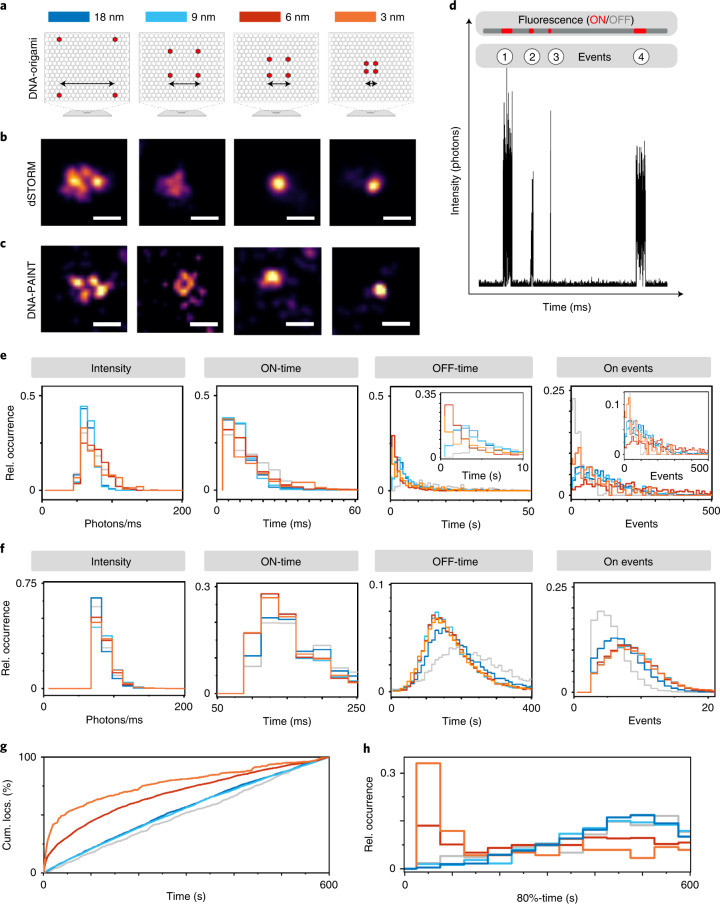
dSTORM and DNA-PAINT imaging of DNA origami. **a**, Scheme of DNA origami labeled with four Cy5 at interfluorophore distances of 18 nm, 9 nm, 6 nm and 3 nm. **b**,**c**, Selected dSTORM (**b**) and DNA-PAINT (**c**) images of DNA origami. Samples were measured 3–5 times independently. Scale bars, 40 nm. **d**, Analysis of fluorescence trajectories recorded from individual DNA origami imaged using 640 nm excitation at an intensity of 5 kW cm^−2^. **e**,**f**, Relative occurrence of fluorescence intensity ms^−1^ in the on-state (Intensity), lifetime of the on-state (On-time), lifetime of the off-state (Off-time) and number of on-states (On-events) detected for DNA origami with different interfluorophore distances in dSTORM (**e**) calculated from *n* = 3–5 and DNA-PAINT (**f**) calculated from *n* = 2–3 individual experiments. Color code; singly labeled reference (gray), 18 nm (dark blue), 9 nm (light blue), 6 nm (red) and 3 nm (orange). **g**, Number of on-events (cumulative localizations, cum. locs.) detected per frame as a function of time during 10 min dSTORM videos (Supplementary Videos 6–11) of DNA origami with different interfluorophore distance (*n* = 3–5). **h**, Histogram of the times after which 80% of all localizations were detected per individual DNA origami (*n* = 3–5).

**Extended Data Fig. 1:**
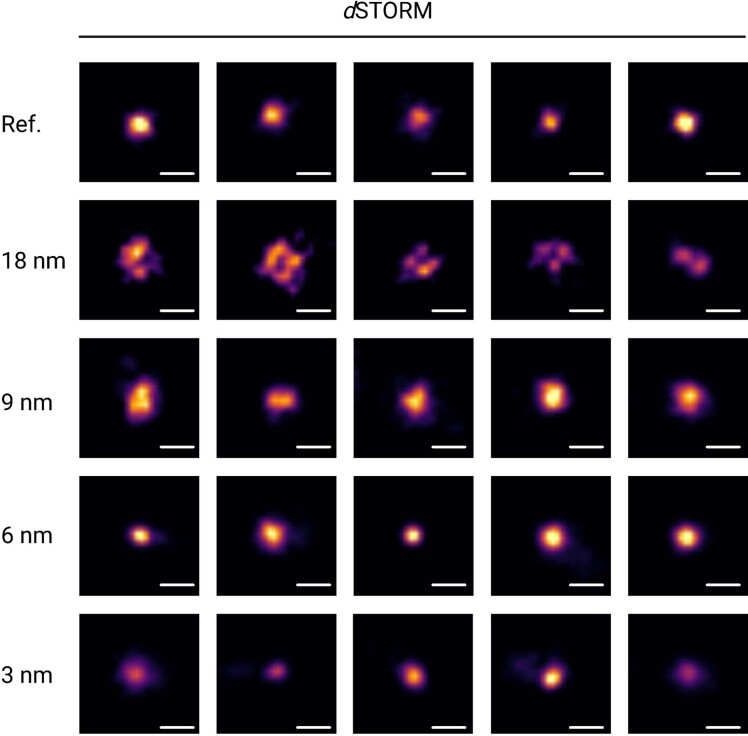
dSTORM imaging of DNA origami. Example dSTORM images of DNA origami with one (reference) or four Cy5 dyes with interfluorophore distances of 18, 9, 6, and 3 nm. Samples were measured 3 – 5 times independently. Scale bar, 40 nm.

**Extended Data Fig. 2:**
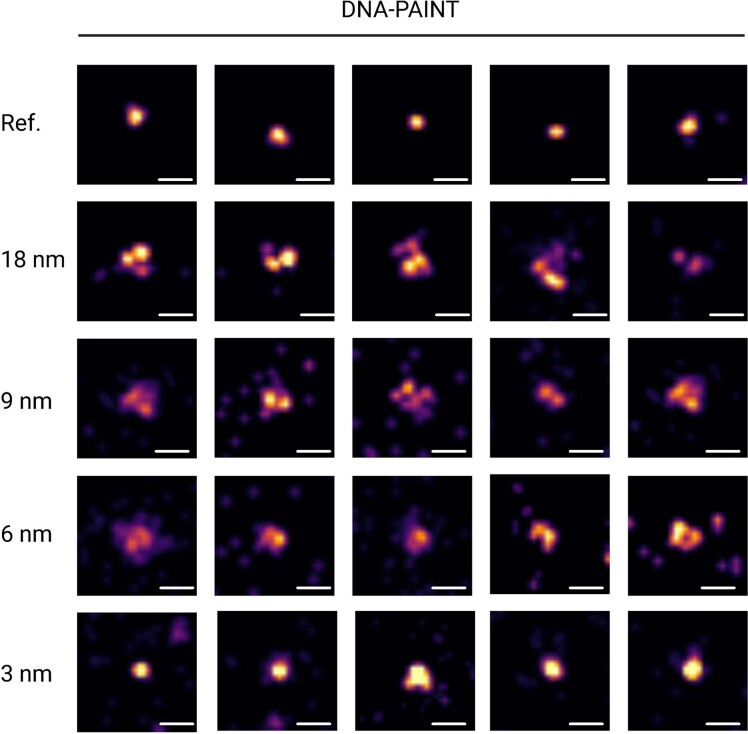
DNA-PAINT imaging of DNA origami. Example DNA-PAINT images of DNA origami with one (reference) or four docking strands separated by 18, 9, 6, and 3 nm. Samples were measured 3 – 5 times independently. Scale bars, 40 nm.

To investigate the problems associated with sub-10-nm fluorescence imaging in more detail, we designed DNA origami^16,17^ carrying four Cy5 dyes separated by 18, 9, 6 and 3 nm and immobilized them on coverslips via biotin-streptavidin binding (Fig. 1a and Supplementary Figs. 1 and 2). As reference we used the same DNA origami labeled with only a single Cy5. dSTORM imaging was performed in standard photoswitching buffer using exclusively 640 nm of irradiation. While in some cases dSTORM can resolve the four fluorophores at an 18 nm distance, it cannot resolve the fluorophores separated by 9, 6 and 3 nm (Fig. 1b and Extended Data Fig. 1). For direct comparison we performed DNA-PAINT using Cy3B-labeled imager strands (Fig. 1c and Extended Data Fig. 2)^18^. DNA-PAINT clearly achieves a higher spatial resolution but also fails to resolve the fluorophores for shorter distances of 6 and 3 nm. Even though we detected intact DNA origami carrying four fluorophores (Fig. 1b,c) we point out that for most DNA origami investigated, we could not detect four fluorophores (Extended Data Figs. 1 and 2). What struck us more, however, is the peculiar difference in photoswitching kinetics, that is, blinking noticeable in the dSTORM videos recorded from the 6 nm and 3 nm DNA origami. While the DNA-PAINT videos recorded for the different origami do not show any difference in blinking behavior throughout the entire recording time (Supplementary Videos 1–5), the dSTORM videos of the 6 nm (Supplementary Video 9) and 3 nm origami (Supplementary Videos 10 and 11) show often a ‘flickering’ fluorescence intensity during the first seconds, that is, very fast blinking compared to the expected well-defined blinking of Cy5 dyes as observed for the reference (Supplementary Video 6) and the 18 nm origami (Supplementary Video 7). Comparison of the fluorescence signal densities during the first seconds and after a few minutes clearly proves that faster blinking is accompanied with faster photobleaching (irreversible destruction of the fluorophore). These observations point out that the on/off photoswitching kinetics of Cy5 dyes is substantially accelerated at shorter interfluorophore distances.

To understand what causes the changes in photoswitching kinetics we have to revisit the dSTORM switching mechanism. dSTORM temporally separates the fluorescence of individual organic dyes by transferring most of them into a nonfluorescent off-state at the beginning of the experiment on irradiation with intensities of a few kW cm^−2^ in thiol-based photoswitching buffer^6,7^. The fluorescent on-state of a small subset of fluorophores is then generated by irradiating the sample usually at shorter wavelengths, that is, typically at 405 nm. Unfortunately, there is no real consensus as to the origin of photoswitching of the two favorite dSTORM cyanine dyes AF647 and Cy5 (refs. ^19,20^). While a recent study identified the formation of a Cy5-thiol adduct with absorption maximum at 310 nm as off-state in dSTORM experiments^21^ another study proposed the formation of a radical formed by one-electron reduction of the cyanine dye absorbing at roughly 450 nm (ref. ^22^). But, Cy5 fluorescence can be restored from the off-state also on irradiation with red light demonstrating that the off-state exhibits a very broad absorption spectrum^21^. This also corroborates the experimental finding that dSTORM imaging can be performed using exclusively irradiation at the absorption maxima of AF647 and Cy5 (refs. ^2,11^).

These considerations indicate that for interfluorophore distances <10 nm the on-state of one fluorophore can serve as donor and excite fluorophores residing in their off-state (acceptors) via fluorescence resonance energy transfer (FRET)^23,24^ into higher excited states from which the on-state can be repopulated. Because FRET from a donor with emission maximum at roughly 670 nm to an acceptor with a low extinction coefficient in the red wavelength range is inefficient, it is difficult to detect by standard means, for example fluorescence quenching of the donor (on-state). However, with increasing number of off-states present in the near-field of a donor the impact of energy transfer on the fluorescence behavior of the multichromophoric system increases and will be measurable. Each FRET event that transfers a fluorophore from the off- to the on-state will change the blinking pattern of the multichromophoric systems. Therefore, we hypothesized that photoswitching kinetics should directly report about the interfluorophore distance in the sub-10-nm range. In practice this means that photoswitching should result in accumulation of fluorophores in their on-state.

Multichromophoric systems with interfluorophore distances shorter than 10 nm show very complex fluorescence trajectories including collective off-states and different intensity levels also in the absence of photoswitching buffer because the fluorophores can interact by various energy transfer pathways including energy hopping, singlet–singlet- and singlet–triplet-annihilation. Therefore, multichromophoric systems often behave like single emitters in photon antibunching experiments^25–27^. In addition, red-absorbing cyanine dyes such as Cy5 show a peculiar complicated behavior because of photoinduced isomerization from the fluorescent *trans* to a nonfluorescent *cis* state and back-isomerization^28^. Furthermore, both the absorption spectra with absorption maximum (*λ*_max_) and extinction coefficient (*ε*) of the triplet state (*λ*_max_ = 695 nm, *ε* = 105,000 cm^−1^ M^−1^) and *cis* state (*λ*_max_ = 675 nm, *ε* = 326,000 cm^−1^ M^−1^) overlap strongly with the fluorescence emission of Cy5 (ref. ^21^). Considering the fact that Cy5 spends roughly 50% of the time in its *cis* state under equilibrium conditions in aqueous solutions that exhibits a lifetime of roughly 200 µs (refs. ^21,28^) it becomes obvious that Cy5 fluorophores separated by less than 10 nm can interact by various energy transfer pathways that result in the observation of blinking processes on different time scales^21,25–27^.

### Consequences for sub-10-nm fluorescence imaging

To explain how the described energy transfer processes compromise dSTORM in the sub-10-nm range, we analyzed the fluorescence trajectories recorded from the different DNA origami at a temporal resolution of 5 ms (Fig. 1d). Analysis of the blinking pattern of individual DNA origami measured in photoswitching buffer, termed in the following photoswitching fingerprint analysis, revealed that the on-state of multiple labeled DNA origami shows similar lifetimes (on-time) but shorter off-state lifetimes (off-times) compared to the single dye reference as expected for multiple blinking fluorophores (Fig. 1e). In addition, the data clearly point out that the off-times decrease with decreasing interfluorophore distance (Fig. 1e), that is the photoactivation rate increases with decreasing interfluorophore distance as expected for energy transfer between the on- and off-states. Furthermore, the on-state intensity is identical to the reference and the 18 and 9 nm DNA origami but slightly higher for the shorter interfluorophore distances (Fig. 1e). Photoswitching fingerprint analysis of data recorded in the presence of glucose-oxidase showed similar results with slightly prolonged off-times (Supplementary Fig. 3a). Since the number of on-events detected with glucose-oxidase is lower (Fig. 1e and Supplementary Fig. 3a) we used standard photoswitching buffer without glucose-oxidase in the following experiments. In contrast, DNA-PAINT experiments revealed photoswitching fingerprints independent of the distance of docking strands. Only the singly labeled reference shows, as expected, longer off-times and fewer on-events (Fig. 1f). This result is expected since the binding of imager to docking strands is sequential in time and decoupled from irradiation.

The low localization probability in both, dSTORM and DNA-PAINT experiments, that is the low number of intact DNA origami detected might be explained by incomplete incorporation of modified oligonucleotides and labeling, respectively. In addition, in DNA-PAINT steric hindrance of docking and imager strands with lengths of 11 and 10 bases can distort labeling and transient binding especially at shorter distances. In dSTORM experiments, fast blinking observed as flickering at the very beginning of irradiation (Supplementary Videos 9–11) promotes fast photobleaching and might thus impede the localization of fluorophores.

**Extended Data Fig. 3:**
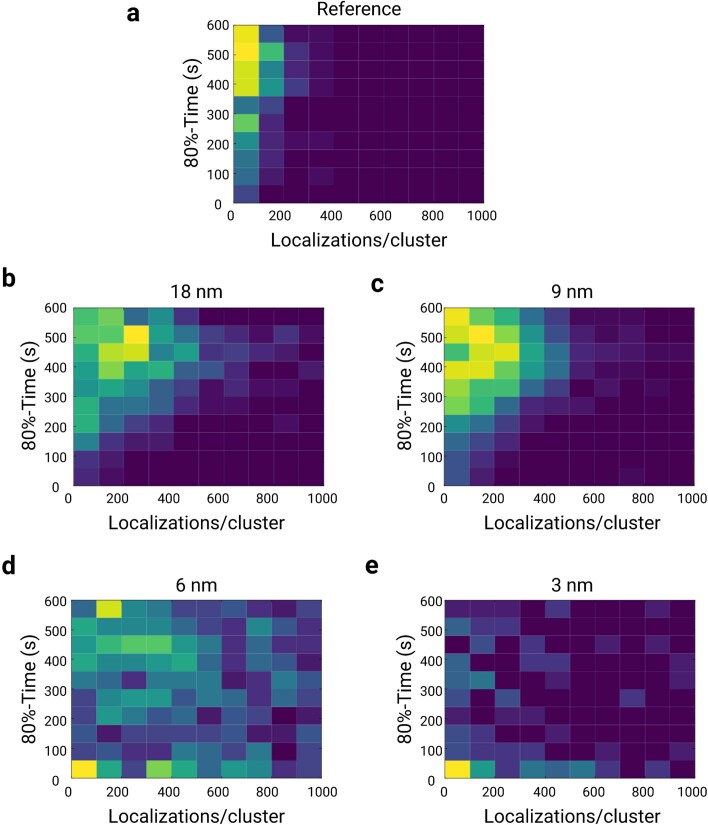
Interfluorophore distances <10 nm are encoded in photoswitching kinetics. Bivariate histograms of detected localizations and the times after which 80% of all localizations were detected per individual DNA origami (n = 3–5). The histograms clearly show that for shorter interfluorophore distances of 3 and 6 nm 80% of all localizations are detected during the first minute.

Another way of looking at the blinking statistics of DNA origami is to plot the summed up localizations detected per frame as a function of time. Here we see that the singly labeled reference and the 9 and 18 nm DNA origami show a linear increase in the number of localizations with time (Fig. 1g and Supplementary Fig. 3b). In a DNA origami carrying a single or four noncommunicating Cy5 dyes each fluorophore will reside on average for several milliseconds in the on-state and several seconds in the off-state. Accordingly, homogeneous blinking is observed until photobleaching occurs. At shorter distances, however, the DNA origami show substantially faster blinking due to energy transfer from the on- to the off-state and subsequent repopulation of the on-state. The distribution of times after which 80% of all localizations are detected per DNA origami confirms fast blinking during the first minutes for most of the 3 and 6 nm DNA origami (Fig. 1h and Extended Data Fig. 3). Thus, our data clearly show that the temporal development of localizations detected from a sample labeled with Cy5 fluorophores changes at interfluorophore distances of <10 nm.

The consequences of our findings for sub-10-nm fluorescence imaging are apparent considering that at the very beginning of a dSTORM experiment all fluorophores reside in their fluorescent on-state and have to be transferred to their off-state on irradiation. Since each Cy5 fluorophore exhibits only a limited number of on/off photoswitching cycles repopulation of the on-state due to energy transfer from the on- to the off-state will accelerate blinking and photobleaching during the first few tens of seconds of the experiment, for example during sample alignment. Consequently, this results in substantially decreased localization probabilities and lower structural resolutions just as in previous experiments^8,12^.

### Time-resolved detection reveals interfluorophore distances

**Fig. 2:**
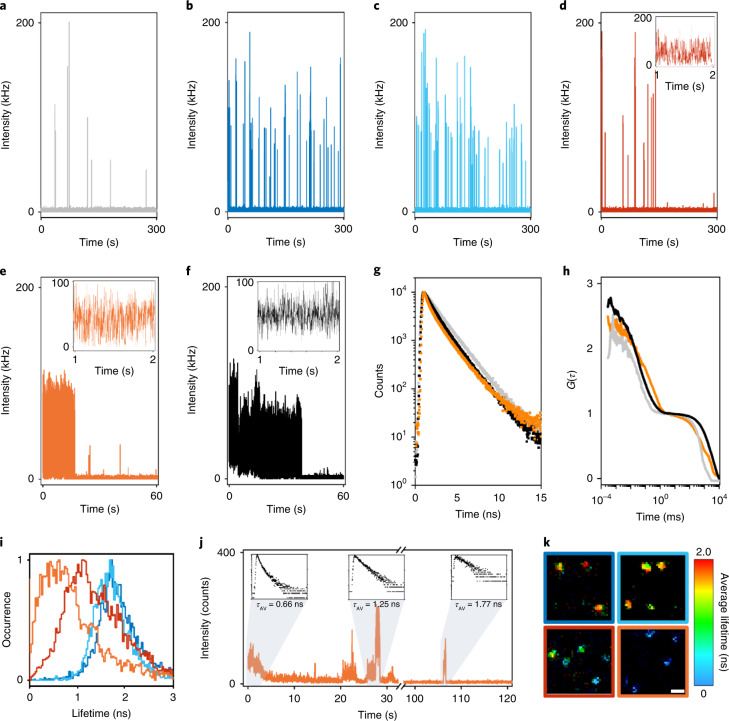
Various energy transfer pathways are responsible for fast blinking observed in the sub-10-nm range. **a**–**e**, Fluorescence trajectories recorded for a singly labeled reference (**a**), 18 nm (**b**), 9 nm (**c**), 6 nm (**d**) and 3 nm (**e**) DNA origamis in dSTORM photoswitching buffer. Color code, singly labeled reference (gray), 18 nm (dark blue), 9 nm (light blue), 6 nm (red) and 3 nm (orange). Zoomed-in trajectories of the first seconds show fast blinking observed for the 6- and 3-nm DNA origamis. Time bins, 1 ms. **f**, Fluorescence trajectory recorded for a 3-nm DNA origami in trolox buffer and zoomed-in fluorescence signal of the first 2 s. Time bin, 1 ms. **g**, Average fluorescence decays from *n* = 7–10 individual fluorescence trajectories of singly labeled reference (gray) and 3-nm DNA origamis measured in trolox (black) and photoswitching buffer (orange), respectively, revealing different energy transfer pathways between the Cy5 fluorophores. **h**, Average intensity autocorrelation functions (G(*τ*)) calculated from *n* = 7–10 individual fluorescence trajectories of singly labeled reference and 3-nm DNA origamis measured in trolox and photoswitching buffer, respectively, normalized to 1 ms. **i**, Histogram of average FLIMs measured from *n* = 7–15 fluorescence trajectories of individual DNA origami with different interfluorophore distances of 18, 9, 6 and 3 nm in photoswitching buffer. **j**, Fluorescence trajectory of a 3 nm DNA origami in photoswitching and corresponding fluorescence decays with average fluorescence lifetimes (*τ*_AV_) of 0.66, 1.25 and 1.77 ns recorded during the gray marked areas. **k**, Typical FLIM images of the 18-, 9-, 6- and 3-nm DNA origami measured in trolox buffer emphasize the increased blinking and shorter fluorescence lifetime of Cy5 fluorophores in the sub-10-nm range (moving from top left to the bottom right). The samples were measured 5–10 times and excited at 640 nm with 2.5 kW cm^−2^ at an integration time of 5 µs pixel^−1^. Scale bar, 1 µm.

To obtain a more detailed picture of the underlying photophysics, we investigated individual DNA origami with higher temporal resolution by time-resolved confocal single-molecule fluorescence microscopy (Fig. 2a–e). Single-molecule surfaces were scanned with very low irradiation intensity to minimize premature photobleaching, individual DNA origami selected and parked in the laser focus to record the fluorescence intensity and lifetime with time. In photoswitching buffer, the reference DNA origami displayed blinking with short (milliseconds) on-states and long (seconds) off-states (Fig. 2a and Supplementary Fig. 4). As expected for four independently emitting fluorophores, 18-nm DNA origami displayed more switching events per time with similar intensities (Fig. 2b and Supplementary Fig. 5). The 9 nm DNA origami showed very similar behavior; however, some parts of the trajectories indicated faster blinking especially at the beginning of irradiation (Fig. 2c and Supplementary Fig. 6). By contrast, some of the 6 nm and 3 nm DNA origami showed clearly very fast blinking at the very beginning of the trajectories. Magnified views of the first seconds of the trajectories emphasize that the off-state lifetimes of DNA origami with interfluorophore distances of 3 and 6 nm are much shorter, that is, blinking occurs on the millisecond time scale (Fig. 2d,e and Supplementary Figs. 7 and 8).

**Extended Data Fig. 4:**
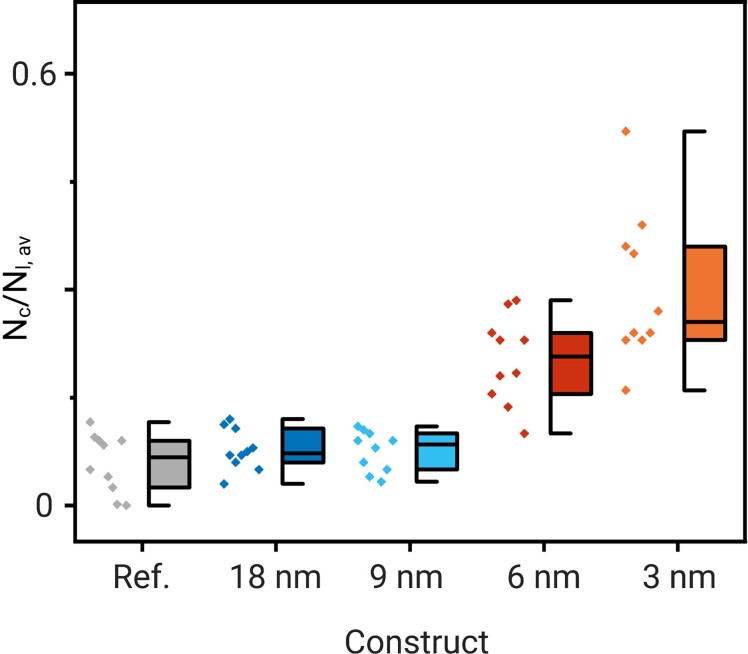
Photon antibunching experiments demonstrate that the on-state is repopulated via energy transfer at interfluorophore distances <10 nm. Photon antibunching experiments demonstrate that the on-state is repopulated via energy transfer at interfluorophore distances <10 nm. Nc/Nl,av ratios measured for n = 10 single-molecule trajectories of the different DNA origami constructs in photoswitching buffer. The box plot values for minima, maxima, mean, percentile 25, 50 and 75 are 0,000, 0.116, 0.059, 0.025, 0.067 and 0.090 (Ref.), 0.030, 0.120, 0.077, 0.060, 0.072 and 0.107 (18 nm), 0.033, 0.110, 0.075, 0.050, 0.085 and 0.100 (9 nm), 0.100, 0.285, 0.202, 0.155, 0.207 and 0.240 (6 nm), 0.160, 0.520, 0.299, 0.230, 0.255 and 0.360 (3 nm).

In addition, we performed photon antibunching experiments to investigate the number of emitting fluorophores contributing to the detected fluorescence signal per single DNA origami (Supplementary Figs. 4–8). Photon antibunching experiments take advantage of the fact that the probability of emitting two consecutive photons drops to zero for a single emitter for time intervals shorter than the excited-state lifetime. For sufficiently short laser pulses, the number of photon pairs detected per laser pulse can be used to determine whether the emission is from one or more independently emitting quantum systems^25–27,29^. Since the intensity of the central peak contains information about the number of independently emitting molecules, the number of photon pairs detected in the central peak, *N*_c_, at delay time zero, to the average number of the lateral peaks, *N*_l,av_, can be used to determine the number of independently emitting fluorophores. For example, neglecting background, *N*_c_*/N*_l,av_ ratios of 0.0, 0.5, 0.67 and 0.75 are expected for 1–4 independently emitting fluorophores^25–27,29^. If, for interfluorophore distances of <10 nm the on-states are repopulated via energy transfer we expect a higher *N*_c_*/N*_l,av_ ratio measured for the 3 and 6 nm DNA origami. And, in fact, the *N*_c_*/N*_l,av_ ratios increase from 0.067 (reference) via 0.073 (18 nm) and 0.085 (9 nm) to 0.207 (6 nm) and 0.255 (3 nm) for the different DNA origami (Extended Data Fig. 4) in accordance with the slightly higher fluorescence intensities recorded in dSTORM experiments form the 3 nm and 6 nm DNA origami (Fig. 1e). Higher *N*_c_*/N*_l,av_ ratios are prevented by efficient energy hopping, singlet–singlet- and singlet–triplet-annihilation between Cy5 fluorophores in the on-state^25–27^.

To dissect the two different energy transfer pathways (*trans*/*cis* and on/off) we investigated the 3 nm DNA origami in the absence of photoswitching, that is in PBS, pH 7.6 containing 1 mM trolox/troloxquinone and an oxygen scavenging system to prolong the observation time^30^. Fluorescence trajectories of the 3 nm DNA origami showed similar blinking behavior in trolox buffer during the first seconds of irradiation but more fluorescence intensity levels (Fig. 2f and Supplementary Fig. 9). The similarity of the trajectories in the absence and presence of photoswitching buffer corroborates our hypothesis that in dSTORM experiments with interfluorophore distances of <10 nm all fluorophores are efficiently transferred to the on-state resulting in fast blinking. Fluorescence decays of individual DNA origami recorded in trolox buffer clearly revealed a fluorescence quenching pathway at 3 nm interfluorophore distance. While the reference DNA origami labeled with a single Cy5 displayed a monoexponential fluorescence lifetime of roughly 1.8 ns, the fluorescence decay of the 3 nm DNA origami exhibited multiexponential kinetics with a short lifetime component of roughly 600 ps due to energy transfer from one fluorophore in the *trans* to another fluorophore residing in the *cis* state, neglecting energy transfer to the shorter-lived triplet state (Fig. 2g). As a photoinduced process, the efficiency of *trans*/*cis* isomerization, that is the degree of energy transfer, is determined by the irradiation intensity and thus not seen in standard ensemble time-correlated single-photon counting (TCSPC) experiments where low irradiation intensities are usually applied (Supplementary Fig. 10).

**Extended Data Fig. 5:**
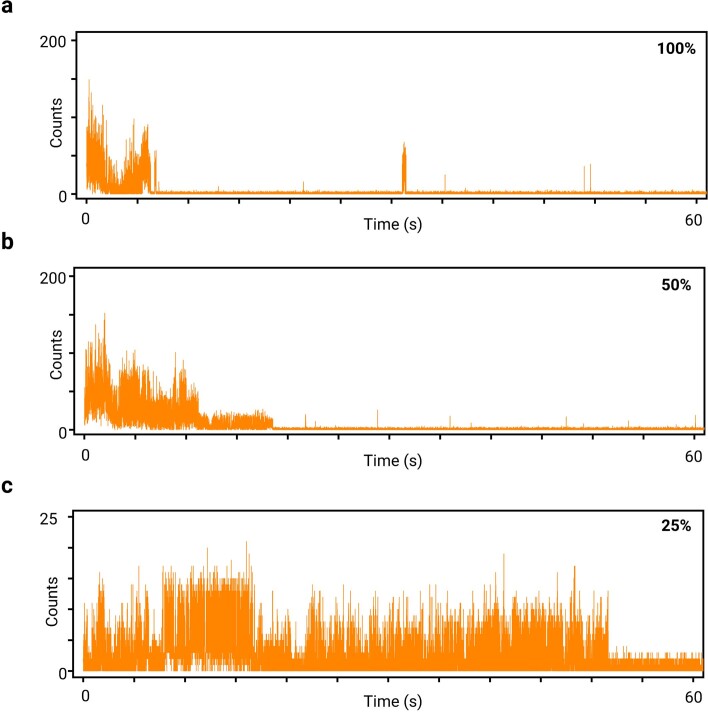
Photoswitching kinetics is slowed down by lower irradiation intensity. Typical fluorescence trajectories of 3 nm DNA origami measured by single-molecule sensitive confocal fluorescence microscopy in photoswitching buffer excited at 640 nm with different irradiation intensities (n = 3). **a**, 100% laser power. **b**, 50% laser power. **c**, 25% laser power (1 ms binning). 100% correspond to 2.5 kW cm^−2^.

The fluorescence decay of the 3 nm DNA origami recorded in photoswitching buffer exhibited multiexponential kinetics with a shorter fluorescence lifetime component of roughly 400 ps (Fig. 2g). The shorter fluorescence lifetime component confirms the additional energy transfer pathway in photoswitching buffer from one fluorophore in the on-state (donor) to another fluorophore in the off-state (acceptor). Furthermore, direct comparison of the fluorescence intensity autocorrelation functions of 3 nm DNA origami recorded during the first seconds of the trajectories demonstrates that the fluorescence fluctuations are dominated by energy transfer from the fluorescent *trans* to the nonfluorescent triplet and *cis* state of Cy5 in trolox buffer. However, in photoswitching buffer an additional small on/off component appears in the few hundred microseconds range that we attribute to energy transfer from the on- to the off-state followed by repopulation of the on-state (Fig. 2h). Since all the observed on/off processes are strongly controlled by the excitation efficiency, reduction of the irradiation intensity slows down the blinking kinetics but simultaneously decreases the fluorescence intensity in the on-state (Extended Data Fig. 5).

In conclusion, the interfluorophore distance determines the off-state lifetime in dSTORM experiments and is also encoded in the fluorescence lifetimes, whereas the number of on-events detected contains information about the number of fluorophores present. Consequently, the fluorescence lifetime of DNA origamis decreases with decreasing interfluorophore distance (Fig. 2i). Furthermore, the fluorescence lifetime of fluorophores in the 3 nm DNA origami increases during the fluorescence trajectory with progressing fluorophore photobleaching (Fig. 2j and Supplementary Fig. 11). This demonstrates that the quenching efficiency of the on-state is determined by the number of off-states (quenchers) present. Fluorescence lifetime imaging microscopy (FLIM) images of the four DNA origami measured in trolox buffer demonstrate that individual 18- and 9-nm DNA origami are imaged with lifetimes of roughly 2 ns, while 6- and 3-nm DNA origami show substantially shorter fluorescence lifetimes (Fig. 2k and Supplementary Fig. 12).

### Sub-10-nm super-resolution fluorescence imaging in cells

To translate our findings into biological applications, that is super-resolution imaging in cells, the labeling problem has to be solved first. While site-specific and efficient labeling of DNA origami with organic dyes is straightforward, site-specific fluorescence labeling of biomolecules separated by only a few nanometers remains challenging. In addition, the displacement of the fluorophore from the point of interest (the linkage error) and the conformational flexibility of the linker determine the localization accuracy achievable in super-resolution imaging experiments. Approaches to minimize the displacement of the fluorophore have been introduced including nanobodies and peptide tags but still yield linkage errors of a few nanometers^31,32^ thus preventing the translation of 1–5-nm localization precision into image resolution in real biological samples. Furthermore, the sheer size of the fluorescent probe including fluorophore, linker and affinity reagent does not only increase the linkage error but also limits the achievable labeling density^33,34^. One approach to solve the labeling problem is direct covalent site-specific attachment of an organic dye to a protein of interest, which can be achieved by GCE incorporating a noncanonical amino acids (ncAAs) into the protein of interest that can be efficiently labeled by bioorthogonal click chemistry with small organic dyes^35,36^. The method enables site-specific efficient labeling of intra- and extracellular proteins with a linkage error of roughly 1 nm with super-resolution microscopy suited organic dyes^37^. Recent studies conclusively demonstrated that click labeling of ncAAs with small tetrazine-dyes is a versatile tool for the labeling of sterically difficult to access protein sites also in crowded environments^38,39^.

**Fig. 3:**
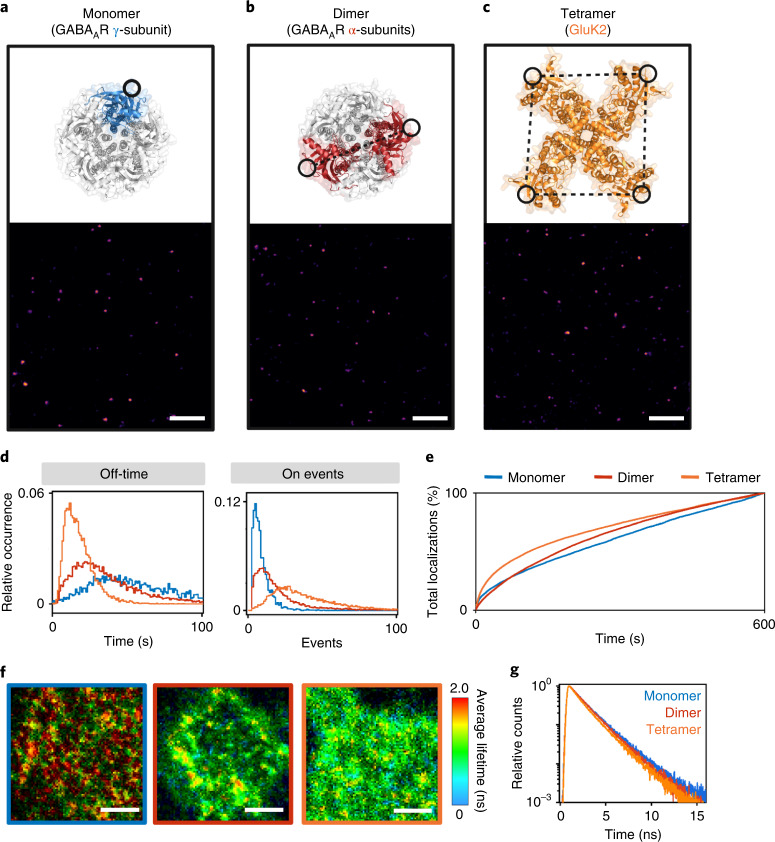
Time-resolved photoswitching fingerprint analysis in cells. **a**–**c**, Molecular structures of the pentameric GABA-A (PDB 6HUG) and tetrameric GluK2 receptor (PDB 5KUF) with incorporation sites of ncAAs shown as black circles (blue, γ2 subunit GABA-A (**a**); red, dimeric α2 GABA-A (**b**); orange, homotetrameric GluK2 (**c**)) and corresponding dSTORM images of HEK293T membrane sections showing fluorescence signals of individual receptors (5 nm pixel^−1^). The ncAAs were labeled by click chemistry with Met-Tet-Cy5. In the GABA-A^S181TAG^ mutant the distance between the two fluorophores in the α2 subunits is roughly 5 nm. In the GluK2^S398TAG^ mutant the distance between the four Cy5 molecules is roughly 7 nm (refs. ^41,42^). The samples were measured 3–5 times independently. Scale bars, 500 nm. **d**, Relative occurrence of lifetimes of the off-state (Off-time), and number of on-states (On-events) detected from individual receptors in dSTORM experiments (*n* = 3–5). **e**, Number of on-events (localizations) detected per frame as a function of time during 10 min dSTORM experiments of membrane receptors (*n* = 3–5). **f**, FLIM images of HEK293T cells expressing monomeric γ2 subunit of GABA-A (left, blue), dimeric α2 subunit of GABA-A (middle, red), and homotetrameric GluK2 receptors (right, orange) click-labeled with Met-Tet-Cy5 measured by confocal TCSPC imaging in photoswitching buffer at an irradiation intensity of 2.5 kW cm^−2^. To minimize photobleaching of fluorophores FLIM images were recorded at 5 µs of integration time per pixel. No intensity threshold was applied. Scale bars, 2 µm. **g**, Average fluorescence decays from *n* = 8–13 FLIM images of HEK293T cells expressing receptors labeled with one, two and four Cy5 fluorophores.

**Extended Data Fig. 6:**
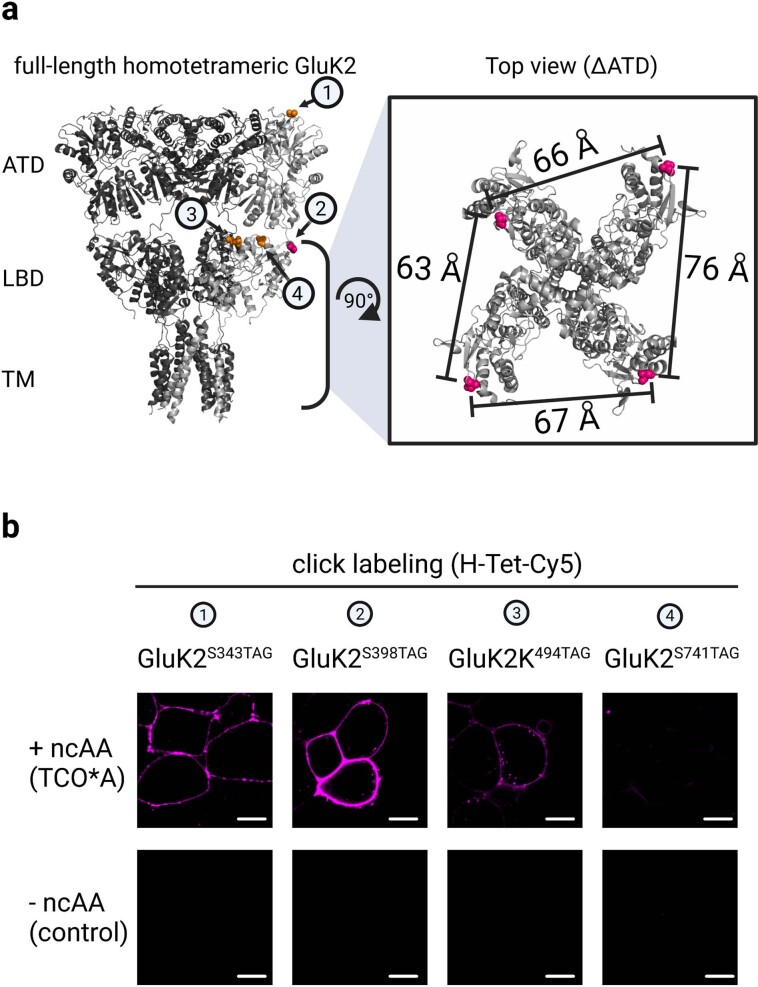
Scheme explaining click labeling and construct design of GluK2. **a**, Different mutants 1–4 were generated within one monomeric subunit of GluK2. Calculation of the distances was performed with PyMOL (Molecular Graphics System, Version 1.2r3pre, Schrödinger, LLC) on basis of the crystal structure (PDB-ID: 5KUF)41,42. **b**, To check the efficiency of ncAA incorporation of the different mutants, click labeling was performed with H-Tet-Cy5. Control experiments without the addition of ncAA resulted in inefficient amber suppression efficiency which leads to premature translation termination and no click labeling. Samples were measured 3–5 times independently. Scale bars, 10 µm.

We hypothesized that the combination of time-resolved photoswitching fingerprint analysis in combination with GCE and click labeling can be used to unravel information about the molecular stoichiometry and interfluorophore distances in the sub-10-nm range in biological samples. We selected two different multimeric proteins, the hetero-pentameric γ-aminobutyric acid type A (GABA-A)^40^ and the tetrameric kainate receptor (GluK2)^41^. Site-specific labeling was achieved by incorporation of one or more *trans*-cyclooct-2-ene (TCO)-modified ncAAs (TCO*-l-lysine) into the extracellular domains of the (1) monomeric γ2 subunit, (2) dimeric α2 subunit of GABA-A and (3) homotetrameric GluK2 (Fig. 3a–c). To identify the best positions for the insertion of ncAAs, various click constructs were generated. All positions were selected to be at unstructured, extracellular regions of the pentameric GABA-A receptor (Protein Data Bank (PDB) ID 6HUG) or homotetrameric GluK2 receptor (PDB 5KUF). The generated click mutants were tested for ncAA incorporation and labeling efficiency in human embryonic kidney 293T (HEK293T) cells (Extended Data Fig. 6).

TCO*-l-lysine (TCO*A) reacts with tetrazine dyes in an ultrafast, specific and bioorthogonal inverse electron-demand Diels–Alder reaction, and allows thus efficient site-specific labeling of receptors with one, two and four Me-Tet-Cy5 dyes, respectively, with minimal linkage error^36–38^. While the interfluorophore distance in the α2 subunits of GABA-A^S181TAG^ is roughly 5 nm, it is roughly 7 nm in the tetrameric GluK2^S398TAG^ (Fig. 3a–c and Extended Data Fig. 6a)^42^. Hence, dSTORM cannot resolve the different fluorophores and the resulting images display homogenous distributions of the receptors with no indications of clustering or different labeling stoichiometry (Fig. 3a–c and Supplementary Fig. 13). Photoswitching fingerprint analysis of the receptor signals recorded in dSTORM experiments shows that the number of on-events and the lifetime of the off-states are unequivocally different reflecting the different number of fluorophores present per membrane receptor. We detected on average roughly 7 and 15 on-events (median) for singly (γ2) and double (α2) labeled GABA-A receptors, respectively, and roughly 33 on-events (median) for the fourfold labeled GluK2 receptor (Fig. 3d). Hence, the number of on-events contains information about the number of fluorophores present per spatially unresolvable area (for example, the receptor stoichiometry) even though the fluorophores are separated by less than 10 nm. This result reports most impressively that site-specific TCO*A incorporation into subunits of multimeric proteins by GCE followed by bioorthogonal click labeling with tetrazine dyes enables quantitative labeling of protein sites separated by only a few nanometers.

The temporal evolution of localizations (on-events) detected per frame displays that energy transfer between the on- and off-states of the four Cy5 fluorophores in the GluK2 receptor results in shortening of the off-state lifetime and correspondingly more frequent blinking during the first minutes of irradiation (Fig. 3e) similar to the observation for the 6- and 3-nm DNA origami (Fig. 1g). For the single and double-labeled GABA-A receptor we could not detect unequivocal differences in the temporal evolution of localizations (Fig. 3e) demonstrating that energy transfer from an on- to a single off-state is hardly detectable in SMLM data at an interfluorophore distance of roughly 5 nm. In the presence of three possible acceptors (GluK2), however, energy transfer to the off-state and repopulation of the on-state can be easily identified (Fig. 3e). FLIM images of HEK293T cells in photoswitching buffer clearly demonstrated that the singly labeled γ2 GABA-A receptor exhibits a monoexponential lifetime of roughly 1.85 ns independent of the irradiation intensity, whereas GluK2 receptors exhibit a shorter fluorescence lifetime because of energy transfer from the *trans* on- to the off- and *cis* state (Fig. 3f and Supplementary Fig. 14). FLIM images of the double-labeled α2 GABA-A receptor are less conclusive confirming that energy transfer from an on- to a single off-state remains difficult to identify even by time-resolved fluorescence spectroscopy (Fig. 3f and Supplementary Fig. 14).

This impression is also supported by average fluorescence decays. Here, both the average fluorescence decays of α2 GABA-A and GluK2 exhibit multiexponential character with short fluorescence lifetime components of roughly 0.65 and 0.75 ns and amplitudes of roughly 0.16 and 0.38, respectively (Fig. 3g). This means that energy transfer between two Cy5 separated by roughly 5 nm at best induces the appearance of a small 650 ps component in the fluorescence decay. On the other hand, energy transfer between four Cy5 separated by roughly 7 nm causes a slightly longer lifetime component of roughly 750 ps but with higher amplitude and is thus far easier to detect.

## Discussion

Since in dSTORM experiments only a single fluorophore is expected to reside in the on-state per diffraction-limited area, being cycled between its singlet-ground and first excited singlet-state for several milliseconds, fluorophore interactions have been presumed to play a negligible role. Hence, a direct relation between fluorophore interactions and image resolution in SMLM experiments is not given. However, as we have shown here dipole–dipole induced energy transfer between the on- and off-states of fluorophores results in faster repopulation of the on-state, and in combination with other additional energy transfer pathways in fast blinking. Such fast switching events are elusive in dSTORM experiments because samples are usually irradiated at high intensity for a while to turn most fluorophores into their off-state before data acquisition. What is more important, however, is the fact that the number of photoswitching cycles and thus localization possibilities of a fluorophore is limited due to photobleaching. If fluorophores show energy transfer-induced fast on/off photoswitching during the first seconds of the experiment, that is during sample alignment, their localization probability decreases substantially. The energy transfer efficiency is controlled by the acceptor concentration, that is the number of fluorophores residing in the off-, *cis* and triplet state. Since these states are populated via the excited state of fluorophores, the observed energy transfer efficiency depends critically on the irradiation intensity (Extended Data Fig. 5 and Supplementary Fig. 14).

The influence of these energy transfer pathways on the achievable spatial resolution in the sub-10-nm range has up to now not been perceived by the super-resolution fluorescence imaging community. Or to be precise, near-field fluorophore interactions that decrease the localization probability of fluorophores separated by less than 10 nm have not been considered so far. The resulting lower image quality sparked a debate about the potential of new structured illumination SMLM methods^8–12^ for molecular resolution imaging^13^ but the contradiction between high localization precision and lower image quality (that is, lower localization probability) remained enigmatic. Our findings are also important for quantitative SMLM approaches. Since photoswitchable fluorophores can repeatedly be localized quantification of molecules is complicated. Multiple methods have been developed to correct for blinking-caused artifacts to enable quantification of SMLM data. However, all approaches rely on interfluorophore distance independent photoswitching kinetics of fluorophores to group localizations that likely come from the same fluorophore^43–45^. Thus, our results clearly identify a new limitation of quantitative SMLM at high labeling densities.

On the other hand, information about the number of fluorophores present and their interfluorophore distances in the sub-10-nm range can be revealed by photoswitching fingerprint analysis. The number of on-events reflects the number of fluorophores, provided that the temporal resolution of the experiment is high enough to resolve fast blinking processes. But this will probably never be the case, since in the initial period where fast blinking occurs too many fluorophores are active to be localized (spatially resolved) individually. The distance between the fluorophores can be estimated from the off-state lifetime (Fig. 1e,d), the temporal evolution of detected localizations (Figs. 1g and 3f), the photon antibunching signature (Extended Data Fig. 4) and the fluorescence lifetime (Figs. 2g,i–k and 3f,g and Supplementary Figs. 11, 12 and 14). For more complex samples exhibiting various interfluorophore distances, for example four fluorophores separated by distances between 2 and 8 nm, photoswitching fingerprint analysis can most likely determine the number of fluorophores present and the shortest interfluorophore distance from the shortest lifetime component. However, all other interfluorophore distances are currently difficult to accurately determine. Since rhodamine and oxazine dyes also enter off-states that absorb at shorter wavelengths and can be photoactivated^20^, photoswitching fingerprint analysis must not remain limited to the red spectral range. The simpler photophysics, that is the absence of *trans*/*cis* isomerization can potentially simplify data analysis. We are currently working on a model that contains all the photophysical processes involved in sub-10-nm fluorophore interactions. A verified theoretical model would allow us to simulate photoswitching fingerprints for arbitrary systems with different fluorophore distances and heterogeneous samples.

Our data show clearly that GCE and site-specific incorporation of ncAAs into proteins followed by click labeling^35–39^ volunteers as method of choice for biological super-resolution microscopy in the sub-10-nm range because of the virtually quantitative labeling efficiency. Anticipating photoswitching fingerprint analysis of endogenous proteins, a potential limitation of GCE with ncAAs is the overexpression of the protein of interest. However, new emergent genome editing tools such as CRISPR–Cas9 might enable site-specific incorporation of ncAAs into endogenous proteins. Furthermore, orthogonal ribosomes^46,47^ in combination with quadruplet codons^48^ will contribute significantly to reduce suppression of endogenous amber codons and improve GCE efficiency, and therefore enable quantitative insertion of multiple ncAA into the protein of interest. Our results also demonstrate that energy transfer between identical fluorophores (for example, between the *trans* and *cis* state and on- and off-state) can be used to determine the interfluorophore distance. Hence two or more identical ncAAs can be incorporated at different sites into the same protein or multiprotein complex. Bioorthogonal click labeling with the same fluorophore in combination with time-resolved single-molecule fluorescence spectroscopy can then be used advantageously for distance measurements akin to standard FRET investigations.

Finally, the disclosure of fast blinking as a result of energy transfer between on- and off-states of fluorophores provides guidance how to further improve sub-10-nm fluorescence imaging. For example, confocal fluorescence lifetime dSTORM^49^ and photoswitching fingerprint analysis might evolve as a powerful super-resolution microscopy method for imaging in the sub-10-nm range. Also, however, the advancement of MINFLUX^8,12^ and especially pulsed interleaved MINFLUX^50^ will benefit from our findings. The only possibility to avoid the acceleration of photoswitching rates and accumulation of dyes in the on-state is the use of DNA-PAINT^18^ where only one imager strand is present per 10 nm area simultaneously during the experiments, or fluorophores whose switching mechanism is independent of irradiation, for example spontaneously blinking dyes such as the Si-rhodamine dye HMSiR^51^ or photoactivatable dyes such as Cy5B (ref. ^52^). Another possibility to avoid energy transfer between adjacent fluorophores is to expand the sample before imaging^53^. Postlabeling expansion microscopy combined with SMLM improves the labeling efficiency and reduces the linkage error thus paving the way for super-resolution fluorescence imaging with true molecular resolution^54^. Although there is ample scope for alternative improvements, the run for an efficient sub-10-nm fluorescence imaging method has just begun.

## Methods

### Design, hybridization and quality control of DNA-origami structures

DNA-origami rectangle structures were designed with caDNAno v.2.2.0 (Supplementary Fig. 1)^55,56^. Stability calculations of the origami designed were performed using CanDO^57,58^. All dye/TCO modified staple strands were ordered at biomers.net GmbH, whereas all biotinylated strands were ordered at Sigma-Aldrich. All unmodified staple strands were ordered at Merck KGaA. We used the phage M13mp18 derivate DNA type p7560 as scaffold DNA (tilibit nanosystems, M1-32). Hybridization was performed by mixing 10 nM scaffold DNA with 15× surplus of unmodified staple strands and 30× surplus of modified staple strands in hybridization buffer, consisting of 5 mM tris(hydroxymethyl)aminomethane (TRIS) (Merck, 1.08382.2500), 5 mM sodium chloride (NaCl) (Sigma, S5880-1KG), 1 mM ethylene diamine tetraacetic acid (EDTA) (Sigma, E1644-250G) and 12 mM magnesium chloride (MgCl_2_) (AppliChem, A4425,0500) using a ThermoCycler (C1000 Thermal Cycler, BioRad) with a linear thermal gradient of −1 °C min^−1^ from 90 to 4 °C. For DNA-PAINT origami, trans-cyclooctene modified staple strands were used. These origami structures were incubated with a tenfold surplus of docking strand 5′-modified with methyl-tetrazine (5′–3′ TTA TAC ATC TA, biomers.net) per TCO-staple for 2 h at 4 °C after hybridization. The hybridized samples were purified by electrophoresis in a 1.5% agarose gel (Sigma, A9539-500G) in 1× TBE buffer, consisting of 4.5 mM TRIS (Merck, 1.08382.2500), 4.5 mM boric acid (Merck, K1898765) and 10 mM EDTA (Sigma, E1644-250G), and 0.5× TBE with 12 mM MgCl_2_ (AppliChem, A4425,0500) as running buffer. After melting the agarose with a microwave, the solution was cooled down to roughly 60 °C until adding 12 mM MgCl_2_ (AppliChem, A4425,0500). Afterward, the gel was poured immediately. A small amount (roughly 10 µl) of a sample was picked as reference, which was mixed with 2 µl intercalating dye (Safe-Green, Applied Biological Materials, G108-G). A small amount of pure scaffold as well as pure staple strands were mixed in hybridization buffer and used as references. These solutions were also mixed with intercalating dye. The rest of the hybridized origami samples were not mixed with intercalating dye. All samples were mixed with loading dye, consisting of 10 mM TRIS (Merck, 1.08382.2500), 60% glycerol (v/v) (Merck, 1.37028.1000) and 0.03% bromophenol blue (w/v) (Carl Roth, T116.1). Electrophoresis was done at 70 V, using a programmable d.c. voltage source (PowerPac Basic, BioRad), for roughly 2 h in water/ice bath. The part of the gel including the references were cut across the length of the gel and the bands marked at an ultraviolet transilluminator (UST20M-8E, INTAS). Afterward, the marked gel was combined with the not illuminated part of the gel, containing the DNA-origami structures not mixed with intercalating dye. Unilluminated DNA origamis were cut out according to the high of the marked references. The extracted gel parts were divided by cutting several times and purified via Freeze N′ Squeeze columns (Freeze N′ Squeeze, 7326165, BioRad) according to the manufacturer’s instructions using a benchtop centrifuge (Biofuge fresco, Heraeus) at 13,000*g*. For all measurements, the DNA origami were produced freshly on the same day of the measurements. The shape and the quality of the purified DNA-origami structures were checked via transmission electron microscopy (JEM 1011, JEOL) and negative staining of the samples (Supplementary Fig. 2). Therefore, carbon coated 100 Mesh TEM-grids were used and glowed freshly. The prepared grids were incubated with 15 µl sample solution for 2 min. Afterward, the solution was peeled of using a filter paper. The grid was dipped into a 0.75% uranyl acetate solution (EMS, 22400) and peeled off immediately. This step was repeated four times until the grid was incubated with 0.75% uranyl acetate solution (EMS, 22400) for 45 s. The solution was peeled off and air-dried.

### Single-molecule DNA-origami surface preparation

For the preparation of DNA-origami single-molecule surfaces, eight chambered cover glass systems with high performance cover glass (Cellvis, C8-1.5H-N) were used. The surfaces were washed once with PBS (Sigma-Aldrich, D8537-500ML) previous treatment with 2% Hellmanex (Hellma, 9-307-011-4-507) for 1 h. After washing the chambers three times with PBS (Sigma-Aldrich, D8537-500ML), the surfaces were incubated with 1 M KOH (Fulka, 06005) for 20 min. After alkaline treatment, the chambers were washed with PBS (Sigma-Aldrich, D8537-500ML). Afterward, the surfaces were incubated with 10% polyethylene glycol 400 (Fulka, 81170) overnight at 4 °C. Afterward, the surfaces were rinsed three times with PBS (Sigma-Aldrich, D8537-500ML) before incubating the chambers with 0.5 g l^−1^ BSA-Biotin (ThermoFisher, 29130) in PBS) overnight at 4 °C. In the following, the chambers were washed three times with PBS (Sigma-Aldrich, D8537-500ML) before incubation with 0.5 g l^−1^ Neutravidin (ThermoFisher, 31050) in PBS (Sigma-Aldrich, D8537-500ML) for 20 min. The surfaces were washed three times with PBS (Sigma-Aldrich, D8537-500ML) and incubated with purified DNA-origami solution, 1:5 diluted in PBS (Sigma-Aldrich, D8537-500ML) + 50 mM MgCl_2_ (AppliChem, A4425,0500) for 10 min. The prepared samples were washed at least three times in PBS (Sigma-Aldrich, D8537-500ML) + 50 mM MgCl_2_ (AppliChem, A4425,0500) before imaging.

### Cell culture

HEK293T cells (German Collection of Microorganisms and Cell Cultures, no. ACC635) were maintained in T25-culture flasks (ThermoFisher, catalog no. 156340) in Dulbeccos’ Modified Eagle’s Medium (Sigma-Aldrich, no. D5796) supplemented with 10% fetal calf serum (Sigma-Aldrich, no. F7524) and 1% penicillin-streptromycin (Sigma-Aldrich, no. P4333).

### Positions for ncAAs insertion

To identify the best positions for the insertion of ncAAs, various click constructs were generated: L198TAG and S217TAG for GABA-A receptors γ2 subunit, K73TAG, S171TAG, S173TAG, S181TAG, S201TAG and K274TAG for GABA-A α2 subunit, and in addition to the previously described constructs S47TAG, S272TAG, S309TAG and S343TAG (ref. ^37^), we tested positions S398TAG, K494TAG and S741TAG for tetrameric GluK2 due to their rectangular positioning.

### Plasmid constructs

All plasmids were amplified by transformation to *E. coli* XL1–Blue followed MIDI-prep DNA isolation and sequencing (Nucleobond, Xtra Midi, Macherey & Nagel, no. 740410). The plasmid for the expression of clickable α2 subunit of the GABA-A receptor was obtained from Addgene (Addgene no. 49169)^59^. The superecliptic pHluorin tag was removed by introducing a XhoI restriction site upstream of the GABA-A coding sequence and subsequent cutting with XhoI–XhoI. The plasmids for the expression of the GABA-AR β1 and γ2 subunits were kindly provided by A. Barberis and described previously^60,61^. The plasmid for the expression of clickable GluK2 was a kind gift from P. Seeburg^62^. The amber stop mutants of GluK2, GABA-AR α2 and GABA-AR γ2 subunits were generated by introducing a TAG stop codon via PCR-based site-directed mutagenesis of the vectors using custom designed primers (Sigma) and Q5 High-Fidelity DNA Polymerase (New England BioLabs). The plasmid for the expression of the tRNA/aminoacyl transferase pair (pCMV tRNAPyl/NESPylRSAF, herein termed PylRS/tRNAPyl) was kindly provided by E. Lemke^63^. The plasmid for the expression of the tRNA/aminoacyl transferase pair (pNEU-hMbPylRS-4xU6M15, herein termed PylRS/4xtRNAPyl) was a gift from I. Coin (Addgene, no. 105830)^64^.

### Transfection of HEK293T cells

Transfection of HEK293T cells was carried out using the JetPrime Transfection Reagent (Polypus, no. 114-01) according to the manufacturer’s instructions. HEK293T cells were seeded on four-well Lab-Tek II chambered glass slides (Nunc, catalog no. 155409) coated with 0.5 mg ml^−1^ poly-d-lysine (Sigma-Aldrich, no. P6407) the day before transfection. At 70–85% confluency the cells were transfected. Transfection of GluK2 receptors was carried out with 500 ng GluK2 and 500 ng pCMV NES-PylRSAF/tRNAPyl per well. GABA-A receptor subunits were transfected at the following ratio with a total amount of 1,750 ng DNA per well: 500 ng α2 subunit, 500 ng β1 subunit, 250 ng γ2 subunit and 500 ng pCMV NES-PylRSAF/tRNAPyl. Additionally, the cells were fed the unnatural amino acid TCO*-A (SiChem, SC-8008) supplemented to the cell media. Therefore, the TCO*-A was diluted 1:4 with 1 M HEPES (pH 8.0) and added at a final concentration of 250 µM to the cells. Transfected cells were maintained in an incubator with 5% CO_2_ at 37 °C for 24 h (GluK2) or 48 h (GABA-AR) depending on transfected constructs and were subsequently labeled with fluorophores.

### Bioorthogonal click labeling of receptors

Transfected HEK293T expressing the TCO*-A modified GluK2, or GABA-A α2 or GABA-A γ2 receptor subunits were labeled with 3 μM tetrazine coupled fluorophores H-Tet-Cy5 (Jena Bioscience, no. CLK-015-05) in cell growth medium for 60 min on ice. Then, cells were washed three times with ice-cold PBS. Next, fixation was carried out with 4% formaldehyde and 0.25% glutaraldehyde for 15 min at room temperature. Following fixation, cells were again washed three times with PBS and subsequently imaged at the dSTORM setup.

### dSTORM and DNA-PAINT imaging

Super-resolution imaging was performed using an inverted wide-field fluorescence microscope (IX-71, Olympus). For excitation of Cy5, a 641 nm diode laser (Cube 640-100 C, Coherent), in combination with a clean-up filter (Laser Clean-up filter 640/10, Chroma) was used. The laser beam was focused onto the back focal plane of the oil-immersion objective (×60, NA 1.45; Olympus). Emission light was separated from the illumination light using a dichroic mirror (HC 560/659; Semrock) and spectrally filtered by a bandpass filter (FF01-679/41-25, Semrock). Images were recorded with an electron-multiplying CCD camera chip (iXon DU-897, Andor). Pixel size for data analysis was measured to 128 nm. For dSTORM measurement, 120,000 images with an exposure time of 5 ms (frame rate 200 Hz) and irradiation intensity of roughly 5 kW cm^−2^ were recorded. Single-molecule surfaces were imaged by epi illumination, whereas prepared cells were imaged by total internal reflection fluorescence microscopy illumination. dSTORM experiments were performed in PBS-based photoswitching buffer containing 100 mM β-mercaptoethylamine (Sigma-Aldrich) and 50 mM MgCl_2_ (AppliChem, A4425,0500) in the absence and presence of a glucose-oxidase-based oxygen scavenger system (5% (w/v) glucose, 10 U ml^−1^ glucose-oxidase and 200 U ml^−1^ catalase) for DNA-origami measurements, or without MgCl_2_ for receptor imaging, adjusted to pH 7.6. For each DNA-PAINT measurement, 18,000 images with an exposure time of 100 ms (frame rate 10 Hz) were recorded. Single-molecule DNA-origami surfaces were imaged by total internal reflection illumination, excited with a 561 nm diode laser (Genesis MX561-500 STM, Coherent) at an irradiation intensity of roughly 1.5 kW cm^−2^ in combination with a clean-up filter (Laser Clean-up filter 561/14, Chroma). Emission light was separated from the illumination light using a dichroic mirror (FF403/497/574-Di01; Semrock) and spectrally filtered by a bandpass filter (BrightLineHC-607/70, Semrock). DNA-PAINT experiments were performed with 5 nM imager strand concentration (5′–3′: CTA GAT GTA T, biomers.net), 5′-modified with Cy3B, in PBS-based buffer containing 5 mM TRIS (Merck, 1.08382.2500), 50 mM MgCl_2_ (AppliChem, A4425,0500), 1 mM EDTA (Sigma, E1644-250G) and 0.05% Tween20 (ThermoFisher, 28320) adjusted to pH 7.6. All SMLM results were analyzed with rapidSTORM3.3 (ref. ^65^) and the highly resolved pictures were reconstructed with ThunderSTORM^66^. The localization precisions were calculated according to Mortensen et al.^4^. For photoswitching fingerprint analysis only, fluorescent spots containing more than 500 (dSTORM)/6,000 (DNA-PAINT) photons per frame were analyzed. To estimate the number of localizations per fluorophore, the tracking function (Kalman filter) of rapidSTORM3.3 was used. Fluorescent spots were tracked over the whole image stack (120,000 frames for dSTORM and 18,000 frames for DNA-PAINT) within a tracking radius of 200 nm. The information was saved as tracked localization file. A custom written python script was used to calculate the number of frames of consecutive localizations per spot (on-time) as well as the number of frames between on-time events of the same fluorescent spot within the defined tracking radius (off-time). In addition, the average number of photons detected per frame as well as the number of on-time events per tracked spot was also calculated.

### Fluorescence lifetime intensity trajectories

All fluorescence lifetime measurements concerning single-molecule trajectories and photon antibunching measurements were performed on a MicroTime200 (PicoQuant) time-resolved confocal fluorescence microscope setup consisting of a FLIMbee galvo scanner (PicoQuant), an Olympus IX83 microscope including an oil-immersion objective (×60, NA 1.45; Olympus), two single-photon avalanche photodiodes (SPAD) (Excelitas Technologies, 75154 K3, 75154 L6) and a TimeHarp300 dual channel board. For pulsed excitation a white-light laser (NKT Photonics, superK extreme) was coupled into the MicroTime200 system via a glass fiber (NKT Photonics, SuperK FD PM, A502-010-110). A 100-µm pinhole was used for all measurements. The emission light was split onto the SPADs using a 50:50 beamsplitter (PicoQuant). To filter out after glow effects of the SPADs used as well as scattered and reflected light, two identical bandpass filters (ET700/75M, Semrock, 294808) were installed in front of the SPADs. The measurements were performed and analyzed with the SymPhoTime64 software (PicoQuant). Measurements were performed with an irradiation intensity of roughly 0.5–2.5 kW cm^−2^ in T3 mode with 25 ps time-resolution, whereas all photon antibunching measurements were performed in T2 mode. For photon antibunching experiments, the Sync cable was disconnected and replaced by the SPAD 2 cable. For analyzing the fluorescence lifetime of the trajectories, the decay parameters were determined by least-squares deconvolution, and their quality was judged by the reduced *χ*^2^ values and the randomness of the weighted residuals (*χ*^2^ of roughly 1). In the case where a monoexponential model was not adequate to describe the measured decay, a multiexponential model was used to fit the decay (*τ*_av_ = *τ*_1_a_1_ + *τ*_1_a_1_). For reference structures and 18-nm DNA origamis, we measured a monoexponential fluorescence decay.

### Photon antibunching measurements

Photon antibunching experiments take advantage of the fact that the probability of emitting two consecutive photons drops to zero for a single emitter for time intervals shorter than the excited-state lifetime. After photon emission, a molecule must be re-excited and wait, on average, one fluorescence lifetime before another photon can be emitted. For sufficiently short laser pulses, the number of photon pairs detected per laser pulse in photon antibunching experiments can be used to determine whether the emission is from one or more independently emitting quantum systems. As expected for dSTORM experiments where only a single fluorophore is expected to reside in the on-state per DNA origami, the ratio of the number of photon pairs detected in the central peak at delay time zero to the average number in the lateral peaks in the interphoton–time (coincidence) histograms is <0.20 demonstrating the presence of a single emitter in the confocal laser focus with low background contributions. This result shows that although increased photoactivation at interfluorophore distances of <10 nm transfers fluorophores from the off- to the on-state the probability for two fluorophores residing simultaneously in the on-state showing independent fluorescence emission is negligible. Even if two fluorophores are simultaneously in the on-state, other energy transfer processes such as homo energy transfer and singlet–singlet annihilation can occur so that the on-state is dominated by the emission of a single fluorophore^25–28^. The data in the interphoton–time histograms can be quantified for the purpose of determining the number of independent emitters by determining the ratio of the number of photons in the central peak, *N*_c_, to the average number in the neighboring lateral peaks, *N*_l,av_. Ensemble antibunching measurements show that the number of photon pairs detected in the neighboring peaks decreases at large interphoton times but is nearly constant for very short times, that is, in the first neighboring peaks. For determination of *N*_l,av_, we used the average number of events in the nearest eight peaks, four to each side of the zero-time peak.

### TCSPC

Measurements take place in a 0.3 mm path-length fluorescence cuvette (Hellma, 105.251-QS) on a FluoTime 200 time-resolved spectrometer (PicoQuant) in combination with a pulsed diode laser (635 nm) as the excitation source with a SepiaII module (PicoQuant), a PicoHarp300 TCSPC module and picosecond event timer (PicoQuant) (80 MHz, 50 ps pulse length, 8 ps resolution, 10,000 photons in the maximum channel). The results were analyzed with the FluoFit v.4.4.0.1 software (PicoQuant). To exclude polarization effects, fluorescence was detected under the magic angle (54.7°). The decay parameters were determined by least-squares deconvolution, and their quality was judged by the reduced *χ*^2^ values.

### Reproducibility

All experiments were performed at least three times. Representative images are shown for each experiment.

### Reporting summary

Further information on research design is available in the Nature Research Reporting Summary linked to this article.

## Online content

Any methods, additional references, Nature Research reporting summaries, source data, extended data, supplementary information, acknowledgements, peer review information; details of author contributions and competing interests; and statements of data and code availability are available at 10.1038/s41592-022-01548-6.

## Supplementary information

Supplementary Information Supplementary Figs. 1–14.

Reporting Summary

Supplementary Video 1 DNA-PAINT videos (30 min) of DNA origami labeled with one (reference) or four docking strands separated by different distances recorded at a temporal resolution of 100 ms per frame. DNA-PAINT of reference DNA origamis, Scale bar, 2 µm.

Supplementary Video 2 DNA-PAINT of 18-nm DNA origamis, Scale bar, 2 µm.

Supplementary Video 3 DNA-PAINT of 9-nm DNA origamis, Scale bar, 2 µm.

Supplementary Video 4 DNA-PAINT of 6-nm DNA origamis, Scale bar, 2 µm.

Supplementary Video 5 DNA-PAINT of 3-nm DNA origamis, Scale bar, 2 µm.

Supplementary Video 6 dSTORM videos (10 min) of DNA origami labeled with one (reference) or four Cy5 dyes with different interfluorophore distance recorded at a temporal resolution of 5 ms per frame. dSTORM of reference DNA origamis, Scale bar, 2 µm.

Supplementary Video 7 dSTORM of 18-nm DNA origamis, Scale bar, 2 µm.

Supplementary Video 8 dSTORM of 9-nm DNA origamis, Scale bar, 2 µm.

Supplementary Video 9 dSTORM of 6-nm DNA origamis, Scale bar, 2 µm.

Supplementary Video 10 dSTORM of 3-nm DNA origamis, Scale bar, 2 µm.

Supplementary Video 11 dSTORM of 3-nm DNA origamis, Scale bar, 2 µm.

## Data Availability

The data that support the findings of this study will be provided by the corresponding author upon reasonable request.

## Extended data

are available for this paper at 10.1038/s41592-022-01548-6.
