# Supplementary material for: Photoswitching fingerprint analysis bypasses the 10-nm resolution barrier
Source: Nat Methods. 2022 Aug 1;19(8):986–94. doi: 10.1038/s41592-022-01548-6 (PMC9349044; doi:10.1038/s41592-022-01548-6)
Supplement: Supplementary file 1 — Supplementary Figs. 1–14. [file 41592_2022_1548_MOESM1_ESM.pdf]

nature portfolio

https://doi.org/10.1038/s41592-022-01548-6

Supplementary information

Photoswitching fingerprint analysis bypasses the 10-nm resolution barrier

In the format provided by the authors and unedited

Supplementary Information

Photoswitching fingerprint analysis bypasses the 10 nm resolution barrier

Dominic A. Helmerich[1†], Gerti Beliu[1,2†], Danush Taban[1], Mara Meub[1], Marcel Streit[2,] Alexander Kuhlemann[1], Sören Doose[1], Markus Sauer[1,2*]

[1]Department of Biotechnology and Biophysics, Biocenter, University of Würzburg, Am Hubland, 97074 Würzburg, Germany

[2]Rudolf Virchow Center, Research Center for Integrative and Translational Bioimaging, University of Würzburg, Josef-Schneider-Str. 2, 97080 Würzburg, Germany

[†]These authors contributed equally

*Corresponding author: m.sauer@uni-wuerzburg.de

Supplementary DNA-origami sequences

All dye modified staple sequences were also ordered unmodified

Start	End	Sequence (5´ - 3´)	modification
10[7]	8[16]	CGAATTCGCCGGGTACCGATAGCATGTCAATCTACCTCGA	
1[56]	3[79]	GTGGATGTTCTTCTAAGTGGTTGTATATCCCATAATCGGC	
9[184]	11[175]	TATAACTACTTAGGTTGGGCACAAGAATTGAGAGAGACTA	
17[184]	19[175]	GTGAGTGAATAAATCAATAGAAACGTCACCAATACCTTTT	
14[71]	18[74]	ATAAATCAAGTACCTTTAATTGCTTTCGGT	3´ - TCO/Cy5 (9nm)
27[8]	27[7]	ATGGTGGTAGAATAGCCCGAGATACCTGTTTG	
23[48]	20[56]	AACTGGCTCATTATACAATCAGGT	
22[71]	25[74]	CATAGTAAAGTATTAAGAG	3´ - TCO/Cy5 (9nm)
2[159]	1[151]	GACGACAATAAACAACGAGCCAGT	
21[80]	16[88]	AATACTGCAAACGAGAATCACCGGAACCAGAGTATAACAG	
31[16]	28[24]	ATTAAAGAACGTGGACTCCCTTAT	
25[80]	20[88]	GACTCCTCACAGTTAAAAGAAAAATCTACGTTAGTTCAGA	
26[167]	29[183]	GGAGGTTTCGTAACGATCTAAAGTTTTGTTAA	
13[24]	10[8]	GTACCAAAGATGAACGGTAATCGTAAAACGCT	
22[63]	20[48]	GAGCAACACTATCATATAATAGTACTTTACCC	
8[175]	11[167]	CAAATCCAATCGCAAGTAGGTCTG	
25[75]	27[79]	GCTGACATTACCCGCTGGCTG	
6[167]	9[183]	TATTCTAAGCTAATATCAGAGAGATAACCTTA	
25[88]	22[80]	AAGAGAAGATAACGCCAAAAGGAA	
25[120]	21[127]	CAGTACCATTAGGAATACCACATTATCTGACAGGAGGTTG	
13[88]	10[84]	CAGTATGTTTTTTGAGAGAT	3´ - TCO/Cy5 (6nm)
30[47]	32[24]	AGACTTTTGGCTACAGCAGCATCGGAACGAGGTCCAACGT	
19[112]	16[120]	CTCCCTCAGAGCCGCCACTAAAGT	
28[183]	28[184]	TTCAGGTTTTTACATCGGGAGAAACGTAGATT	
21[184]	23[175]	AAACATCAGAAGATGATGAAGCCAGAATGGAATTCATTTC	
25[24]	22[8]	TTGGGCTTCGACGATAAAAACCAAAATAGACC	
30[39]	32[48]	TCATGAGGAATTCGACAACTCGTATTAAATCCGCGAAAGA	
5[88]	2[80]	GTGCATCTCTGAACAAGAAAAATA	
31[80]	28[88]	CCAAAAGGAGCCTTTACATGTTAC	
24[175]	27[167]	ACAAAATCGCGCAGAGTACAGTAA	5´ biotin
26[143]	31[135]	GAACCGCCCGTAACACTGTAGCATTCATCGCCGAATTTCT	
14[7]	12[16]	AAGTGTAAGAGCCGGAAGCAGCTAAATCGGTTCGCTCACA	
17[88]	14[80]	TAAGAGGTTAATAGTAGTAGCATT	
14[111]	17[101]	GGAAGGTAAATATTACCATTTT	
21[24]	19[15]	CAAAAGAATCAAATATCGCGTTTTAATTCCCTTAATGAAT	
33[56]	28[48]	TTTGCCCGTTTTCACGAGGACTAAGAAAGAGGAAGGGAAC	
14[159]	12[144]	TTTACCAGCGCCAAAGACGCAAAGAAGCCCTT	
29[56]	25[63]	CAAAAGAAGTAACAAAGCTGCTCACTCTGAAACATGAA	
13[184]	15[175]	AAGACGCTCGATAGCTTAGATCAATAGAAAATCCCTTAGA	
20[175]	23[167]	TACATTTAACAATTTCGCGAATTA	
29[152]	24[144]	ATAGTTAGAGTACCGCCACCCTCACGAGAGGGTACAGGAG	
17[24]	15[15]	AACCAGACCAATAAAGCCTCAGAGCATAAATAGTGCCTAA	
3[144]	0[152]	AGCCGTTTTTATTTTCTTCTTACC	
5[24]	3[15]	GAACAAACAGGGTTTTCCCAGTCACGACGTCTTGGGCACG	
33[0]	32[16]	TTGAGGATTTAGAAGTATTAGACTCAAAGGGC	
6[83]	7[79]	GCACCCAGCTACGCGTCTTT	
23[16]	20[24]	CCCTTCACCGCCTGGCAGAGGCGG	
9[102]	10[102]	AAGCATTAGACCGGAGAGGG	3´ - TCO/Cy5 (6nm)
11[16]	6[16]	TGTTTCCTGTGTGAAAGTGCTTGTATATGTACGTGAGCGAGTAACAAC	
29[88]	26[80]	CCCCAGCGACAAGAACCGGATATT	
5[56]	2[48]	GTCACGTTTACGAGCAAAGGCGAT	

15[48]	12[53]	GTTTAGCTATATTTTCGAAGAGAAGCC	
9[56]	6[48]	CAAAAACACCTGAATCAGCCAGCT	3′ - Cy5 (18nm)
2[135]	5[143]	CTAATGCATCAGGAAGATCGCACT	
29[24]	26[8]	ACTACGAATGCCCTGACGAGAAACACCAGCAG	
21[88]	18[80]	GGAATCGTCGTTTTCATCGGCATT	
31[144]	28[152]	TTGATACCGATAGTTGTACAAACT	
22[39]	25[48]	TTACCAGAGAGATGGTTTAATTTCA	3′ - TCO/Cy5 (9nm)
17[120]	13[119]	TAATTGCTCAACCGATTGAGGGAGTACATACA	
26[111]	29[111]	GCCACCACCCTCATTGATTATACCAAGCGCGA	
18[101]	21[111]	GTAGCGCATAAATATTCATTGA	
15[112]	12[117]	TCATTAAAGGTGAATTAGATAGCCGAA	5′ biotin
6[135]	9[143]	TCCCGACTACACCCTGAACAAAGT	
26[7]	24[16]	GCGAAAATGTTTGCCCCAGAACGAGTAGTAAAAGTTGCAG	
11[8]	11[7]	GTCATAGCATTCCACACAACATACTAATCATG	
33[88]	29[87]	AGTAACATAGGAACAACTAAAGGATCTTTGAC	
33[24]	30[8]	TTACAAACAAGTTTCCATTAAACGGGTAATGT	
3[16]	0[24]	AATATAGGGGCCTTGACGCCCTGG	
31[48]	28[56]	AGGCTTTGTTGAAAATTCAATCAT	
16[79]	19[79]	CAATTCTGCGAACGAGTCTTTTCATAATCAAA	
1[16]	0[0]	AAACGACGAGTGACTCTATGATACCGACAGTG	
4[143]	7[135]	AGCTTTCCGGCACCGCCCAATCCA	
28[111]	31[103]	AATCCGCGACCTGCTCATTGTATC	
22[111]	27[111]	CAACTAATGAACTAACCCTTGAGTAACAGTGCATCTTTTCAGGGATAG	
22[7]	20[16]	AGTGAGACTTTTTCTTTTCCGAGAGGCTTTTGTTTGCGTA	
21[56]	18[48]	AAATGTTTTTATTAGCGATTAAGA	3′ - TCO/Cy5 (18nm/9nm)
3[96]	5[102]	TCCAAGAAGATAAGTCGCCAGTT	3′ - Cy5 (3nm)
5[120]	2[112]	TATCGGCCGAACGCGCCTGTTTAT	
24[79]	27[71]	TGCCCCCTGCCTATTTGCGCATAG	
1[24]	3[47]	GCCAGTGCCAAGCTTTCTCAGGAGTAAGTTGGAAGGGGGA	
7[144]	4[144]	ACGATTTTTTGTTTAAATCGTAGGAATCAGCC	
3[118]	0[120]	AGTACCGCACAGGGCTTA	
5[48]	0[56]	TGGGATAGGCGGGCCTTGTGCTGCTGTAGAAACAAAATAA	
31[168]	32[152]	TACCATATCTGAATAATGGAAGGGCGCCGACA	
7[16]	4[24]	GGGCTTAAGCTACGTGATCGGCTG	
30[143]	32[120]	GGATTTTGTAAACAGCAACCATCGCCCACGCATAACCGAT	
9[80]	4[88]	AAATATTTGCATTAAACCAGAGCCTAATTTGCCGCCATTC	
16[183]	16[184]	GCTTCTGTATCCTTGAAAACATAGATAACCTT	
13[120]	8[112]	TAAAGGTGAGCTGATAAATTAATGCGGGAGAAATAAAAAC	
2[39]	7[47]	GTAACGCCGGCGGATTGACCGTAATTCATCAACGTCTGGC	
1[120]	3[143]	TGTAATTTAGGCAGAGGCATTTTCATGTTCAGAACAAGCA	
4[15]	1[15]	CACATAAATCATTTCTCTCGTCGGGTAAGCAACGGCCCTGCCATTGTA	
1[80]	3[71]	GAATTCATGTCAACCTTATGACAATGTCCCGCCCAATCAA	
18[135]	21[143]	GCGACAGAAGGCAGGTCAGACGAT	
22[143]	25[143]	CCACATCAGTTGAGATGGCGGATAAGTGCCGT	
7[104]	11[103]	AAATAAACAGGGAAGCTATTTTGTAAGGGTGA	
18[39]	23[47]	GAAAGACTGTTTTGCCAGAGGGGGACCCTCGTATTTTAAG	
21[152]	17[159]	ATATTCACTCGATAGCAGCACCGTCTCATCACCAGTAGCA	
8[55]	4[56]	TAACCAATCTTCCTGTTTACCAACCGATCGGT	
22[167]	25[183]	AGCGCAGTAGTATAGCCCGGAATAGGTGTTCG	
11[128]	15[135]	CCATCAATATGATAATTTTAAGAAAAGTAAGCATCACCGT	
26[135]	29[143]	ACCCTCAGGATTTGTATCCACAGA	
5[184]	7[175]	ATCTTCTGTTTTAGTTAATAAGGCTTATCCGGCGCGAGAA	
9[120]	6[112]	TTAACTGATGCGGGAGGTTTTGAA	3′ - TCO/Cy5 (3nm)
28[175]	31[167]	TAACGTCAGATGAATATTAGAACC	
8[47]	11[39]	AGGAACGCCATCAAAAAAATTTTT	
14[39]	19[47]	AAATTAAGCGGAAGCAAACTCCAAGGAAGCCCCGGATTGC	
4[47]	7[39]	CTTCGCTATTACGCCAATAATTCG	5′biotin

28[87]	24[88]	TTAGCCGGACCTTCATCAAGAGTACCGTATAA	
5[112]	3[117]	GACGACAGCCGGAAACAAACCA	5′ - TCO/Cy5 (3nm)
18[127]	16[112]	ATCAAGTTTGCCTTTATTAGAGCTACGGTGTC	
1[88]	3[111]	GCGCACGACTTAAGTGTTTAACAACAACAATACGGGTATT	
25[152]	21[151]	TTGATATACTCTGAATTTACCGTTTGGCCTTG	
8[183]	8[184]	TGCTGATGAACTTTTTCAAATATATATGTAAA	
4[79]	7[71]	CAACTGTTGGGAAGGGGCTAACGA	
2[167]	5[183]	GTCCAGACTAGCAAGCAAATCAGATATAGTTC	
20[15]	19[7]	TTGGGCGCCAGGGTGGAGCTGCAT	
30[79]	32[56]	ATTGCGAAAAAAGGCTTTTGCGGGATCGTCACCCTCAGCA	
22[103]	25[111]	GCAGATACGATTAGGATTAGCGGG	
7[112]	4[120]	AGCCATATTATTTATCTTCTGGTG	
31[112]	28[120]	AGCTTGCTTTCGAGGTTGATAAAT	
26[71]	29[79]	AAATCAACTACACTAAAACACTCA	
20[79]	23[71]	ATGACCATAAATCAAACAGTCAGG	
30[103]	32[112]	GAATAGAATATCATTTGCGGAACAAAGAAACATATTCGG	
23[8]	23[7]	GCTGATTGCAAGCGGTCCACGCTGGGGCAACA	
12[52]	8[56]	TTTATCATATATTTTAAATGCTCATTTTT	
16[23]	12[24]	GCGTTGCGTGAGTGAGCTAACTCATTGTTATC	
30[7]	28[16]	TCCAGTTTGGGTTGAGTGTAATACGTAATGCCAAATCAAA	
10[143]	13[143]	AACATTCAACCGTTCTGCAACATATAAAAGAA	
10[39]	13[47]	AGAGAATCAACATTATGACCCTGT	
24[52]	27[39]	ATTTTAATCATTGTGAATTACGAAAGAGG	
4[175]	7[167]	TTGAAATACCGACCGTACAAAGAA	5′biotin
27[48]	24[53]	ACGGTGTACAGACCAGCGGAACCTATT	5′biotin
32[183]	33[191]	TTATACTTCAAAATTATTTGCACGTGTTTGGAATCCTGAT	
6[143]	8[128]	AGCGAACCAATAAGAAAGCAGCCTTTACAGAG	
10[63]	9[55]	TTGCCTGAGAGTCTGGAAAAGCCC	
1[112]	0[96]	CGCCAACAATTGAGAATCGCCATATCCTTAGT	
13[80]	8[88]	TTATTACGGCAATAATGTAGGTAAAGATTCAATAAAATTC	
14[47]	19[39]	AATTAGCAAATAACCTATTAGATACATTTCGCAAGCAAAG	
18[7]	17[23]	GTCGTGCCCAGTCGGGAAAGAGCTTCAAAGCG	
12[116]	8[120]	CAAAGGGAGACAGTCAAATCAAGAATAAC	
28[119]	24[117]	TGTGTCGACAAGCCCAATAGGAACTTTAACGGGGT	
20[151]	16[144]	CCGCCACCCCCTCAGAGCCACCACTAGAGCCAGCAAAAAC	
30[71]	32[80]	TAATAATTAACGTTATTAATTTTAAAAGTTTGAGGCCGCT	
5[103]	6[102]	TGAGGGGACGCCTTAAATC	3′ - TCO/Cy5 (18nm/6nm/3nm)
10[71]	15[79]	TCAGGTCAGCATGATTAAGACTCCAACATCCACGCGAGCT	
12[183]	12[184]	TCAATAGTCCTTTTTAACCTCCGGGAGAAGAG	
20[47]	23[39]	TGACTATTATAGTCAGCTTATGCG	
19[16]	16[24]	CGGCCAACGCGCGGGGCATTAATT	5′biotin
24[183]	24[184]	ACCAAGTTAATTACCTGAGCAAAACTTTGAAT	
17[80]	12[88]	CCTTTTGATTGATTCCGAAAAGGTGGCATCAAGAGGAAAC	
23[128]	27[135]	AGGTAGAAAGATTGTATGTACTGGTAATAAGTCCATGTAC	
27[144]	24[152]	TGAGTTTCGTCACCAGTTTGATGA	
18[143]	20[128]	AATCAGTAAACCGCCAAGAACCACCACCAGAG	
16[15]	15[7]	CTCACTGCCCGCTTTCAGCCTGGG	
29[184]	30[179]	AGAAATTGTAAAACAGAAACG	
18[167]	21[183]	TGAAACCAAAACAAATAAATCCTCATTAAAAC	
10[83]	11[79]	CTACAAAGGCTAAGTAATGT	
19[48]	16[48]	ATCAAAAAGTTTGCCATAGATTTAGTTTGACC	
9[152]	5[159]	AATTGAGCGAACGCGAGGCGTTTTCCATTACCGCGCCCAA	
8[79]	11[71]	TTTTTGTTAAATCAGCAATGCCTG	
4[111]	7[103]	CAGGCAAAGCGCCATTCAGTTACA	
23[144]	20[152]	AGCGTCATACATGGCTCCTCAGAG	
26[48]	31[39]	ATTCAGTGAACAGATGACGAACTGACCAACTTTGTAGCAAC	
4[183]	4[184]	TTAATGGTTAAATAAGAATAAACAACCTAAAT	

11[144]	8[152]	GAAATAGCAATAGCTACGTCAAAA	
18[73]	23[79]	CATAGCCCCCAGACTGGATAGCGTCCTTACGAGGACGTTGGG	
14[135]	17[143]	GCGACATTGAATATAATGCTGTAG	
25[184]	27[175]	CCTGATTGCAATAACGGATATCACCGTACTCACAGTACCT	
13[152]	8[144]	ACACCACGAATAATAAGAGCAAGACAGAGGGTATGAAAAT	
10[47]	15[39]	AGCAAACAAGAACCCTTTCAACGCAAGGATAAAAATGGTC	
19[104]	23[103]	GGAACCGCCTCAAATGCTTTAAACAATAAAAC	
26[39]	29[47]	ATAAGGCTGGCACCAACCTAAAAC	
1[48]	3[39]	AAGCCAGGCCCCGCTTCTAATCTATTTACGCTGCTGGCGA	
0[151]	3[135]	AGTATAAAGCCAACGCTCAACAGTTCATCGAG	
27[16]	24[24]	TCCGAAATCGGCAAAACCTGAGAG	
30[178]	33[183]	TCTTTCCAGACGTTAGTAAAGATGATGGCAATTCATCAATATA	
1[152]	1[183]	AATAAGAGAATATAAAGTACCGACAAAAGATT	
5[80]	0[88]	TCGTAACCAGGCTGCGTGTCTTTCCTTATCATGCTGAATT	
0[191]	3[175]	AAGCCTGTACTAGAAACCGGAATCATAGTAAAGTAATTCTTAAGGCGT	
6[101]	13[111]	AAGATTAACGTTTAGCTATAGCAAACGTAGAAAA	
14[143]	19[135]	ACAAAAGGCACCGACTATGTTTAAATATGCAACCCTCAG	
0[183]	3[167]	TTAGTATCATATGCGTTATACAAAGTGATAAA	
16[175]	19[167]	AAATCGTCGCTATTAAATTTGAAT	
2[71]	5[79]	TCCTAATTGGTGTAGATGGGCGCA	
30[135]	32[144]	CTAAACAAGGAGCGGAATTATCATCATATTCCATGACAAC	
10[167]	13[183]	TTAAGCCCGAATAAGTTTATTTTGTCACAATT	
6[39]	9[47]	CATTAAATCCCGGTTGATAATCAG	
7[8]	7[7]	AGTAAACATAAAGACGGAGGATCCGTGTAATG	
11[104]	12[96]	GAAAGGCCTTACCAGAAGGAAACC	
29[120]	25[119]	ACAACGGAAACCGCCACCCTCAGAGTTTTGCT	
12[175]	15[167]	GAATTTATCAAAATCATTAATTTT	
30[111]	32[88]	CGGAGTGAGGTTTATCTCGCTGAGGCTTGCAGGGAGTTAA	
33[152]	28[144]	TGATTATCATGAATTTTCTGTATGCAGCCCTCACAACGCC	
17[102]	18[102]	TGCGGATGGCGCGTCAGACT	3′ - TCO/Cy5 (18nm)
24[116]	20[112]	CAGTGGGAACAACATTATTACCCGCCGCCAGCATCCC	
12[79]	15[71]	AACGGAATACCCAAAAATTTGGGG	
31[8]	31[7]	AGTCCACTGAAAAACCGTCTATCAGGAACAAG	
28[79]	31[71]	AACGAGGCGCAGACGGCTCCAAAA	
6[15]	4[16]	CCGTCCCTCCTGGTTGCCGAACTCTGAGGATTCTCCGTGGACGCATTT	
15[144]	12[152]	TGAGCCATTTGGGAATTCTTACCG	
9[88]	6[84]	AAATTGTAAGTTGCTATTTT	3′ - TCO/Cy5 (6nm)
17[48]	13[63]	CAGGTCAGGATTAGAGTACAGGCAAGGCAAAGAATACTTTTGCGGCTG	
20[183]	20[184]	AAATTAATTTAATGGAAACAGTACAGAAAACA	
15[96]	19[103]	TTCTGACGGAAATTATTGGAAGTTTCATTCCACCACCACC	
33[120]	29[119]	CACCAGAACTTTCAACAGTTTCAGAACAAAGT	
14[167]	17[183]	TCATATGGCCATTACCATTAGCAAGGCCGTAT	
6[71]	9[79]	AATTTATGGAAGATTGTATAAGC	
10[7]	8[16]	CGAATTCGCCGGGTACCGATAGCATGTCAATCTACCTCGA	
1[56]	3[79]	GTGGATGTTCTTCTAAGTGGTTGTATATCCCATAATCGGC	

Supplementary Figures

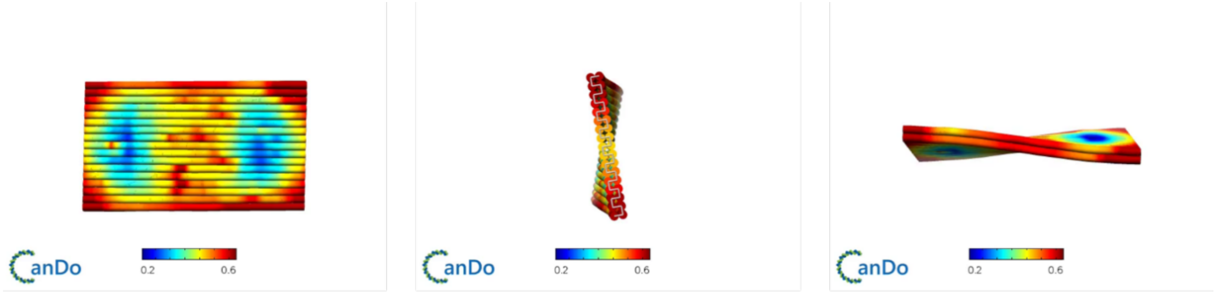

Supplementary Fig. 1. DNA origami stability calculation. The stability of the designed rectangle DNA origami structure were calculated using the open-source software CanDO (*57,58*) with standard DNA parameters. Heatmap in nm.

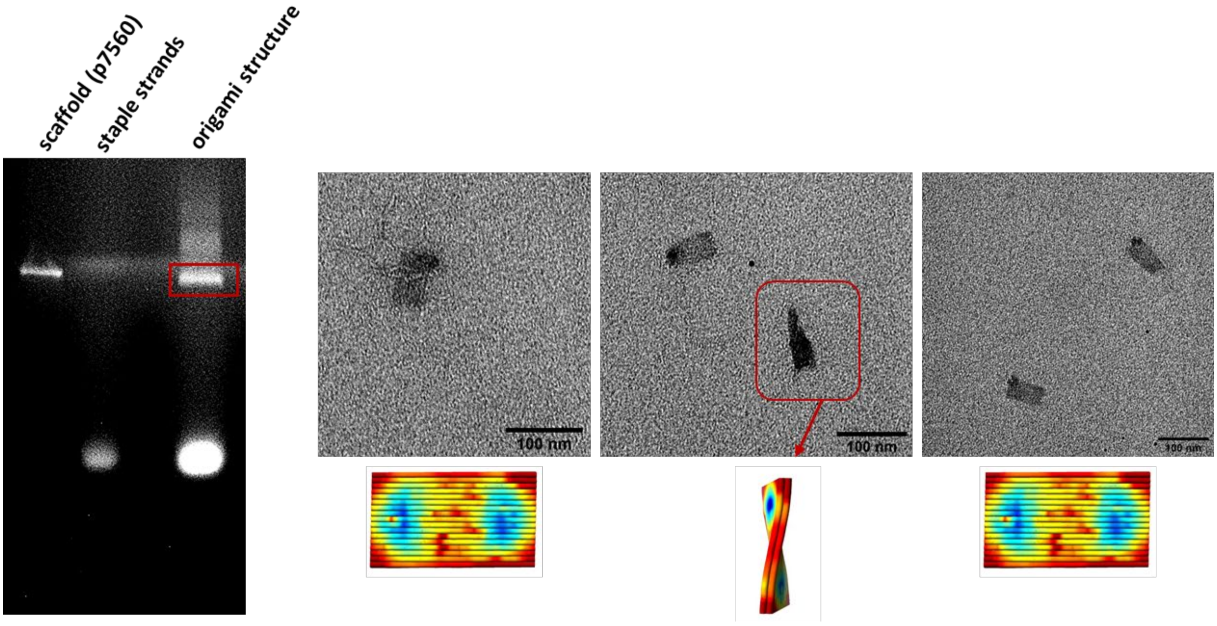

Supplementary Fig. 2. DNA origami quality control. Hybridized DNA origami structures were purified using 1.5% agarose gel. The marked band was cut out and the shape of the DNA origami structures were analyzed using electron microscopy. Samples were measured 10 – 20 times independently.

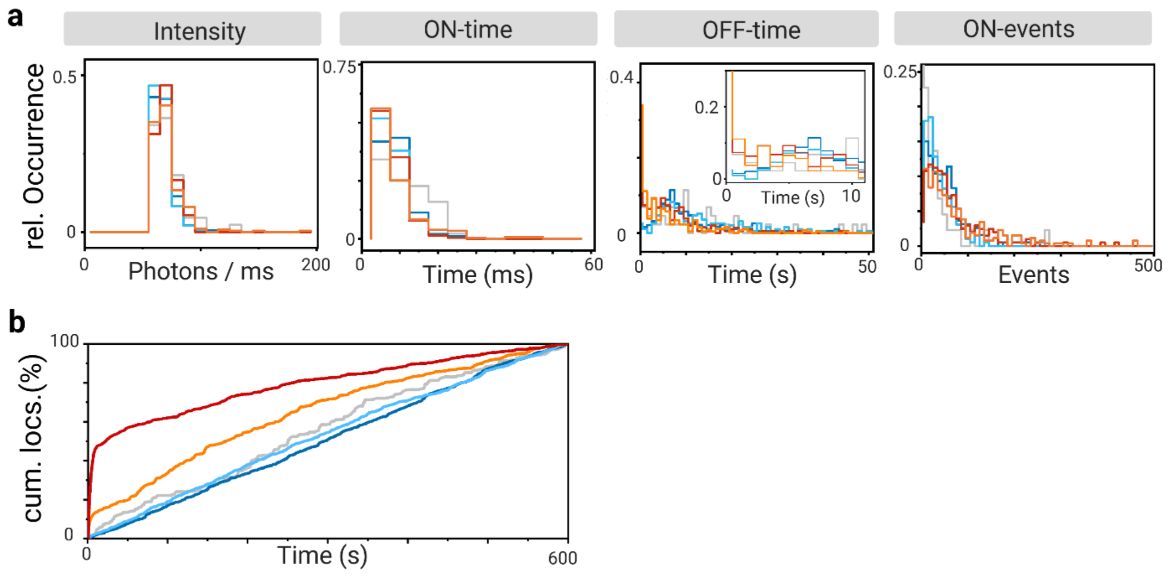

Supplementary Fig. 3. Photoswitching fingerprint analysis of DNA origami imaged by *d*STORM in standard photoswitching buffer in the presence of oxygen scavenger. **a**, Relative occurrence of fluorescence intensity ms^{-1} in the on-state (Intensity), lifetime of the on-state (On-time), lifetime of the off-state (Off-time), and number of on-states (On-events) detected for DNA origami with different interfluorophore distances (n=4) (Color code: singly labeled reference (grey), 18 nm (dark blue), 9 nm (light blue), 6 nm (red), 3 nm (orange)). **b**, Number of on-events (cumulative localizations, cum. locs.) detected per frame as a function of time during 10 min *d*STORM movies of DNA origami with different interfluorophore distance.

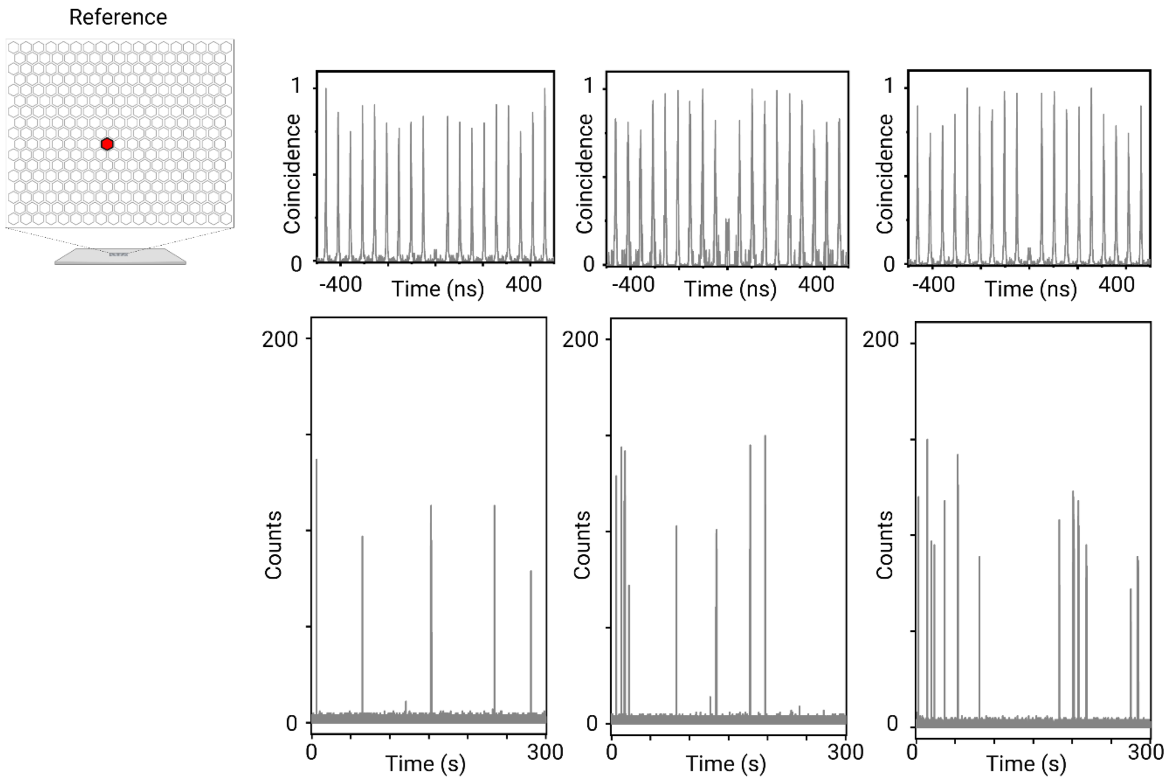

Supplementary Fig. 4. Fluorescence trajectories and corresponding normalized interphoton time (coincidence) histogram measured for the entire trajectory of singly Cy5-labeled reference DNA origami measured by single-molecule sensitive confocal fluorescence microscopy in photoswitching buffer excited at 640 nm with 2.5 kW cm^{-2} (1 ms binning).

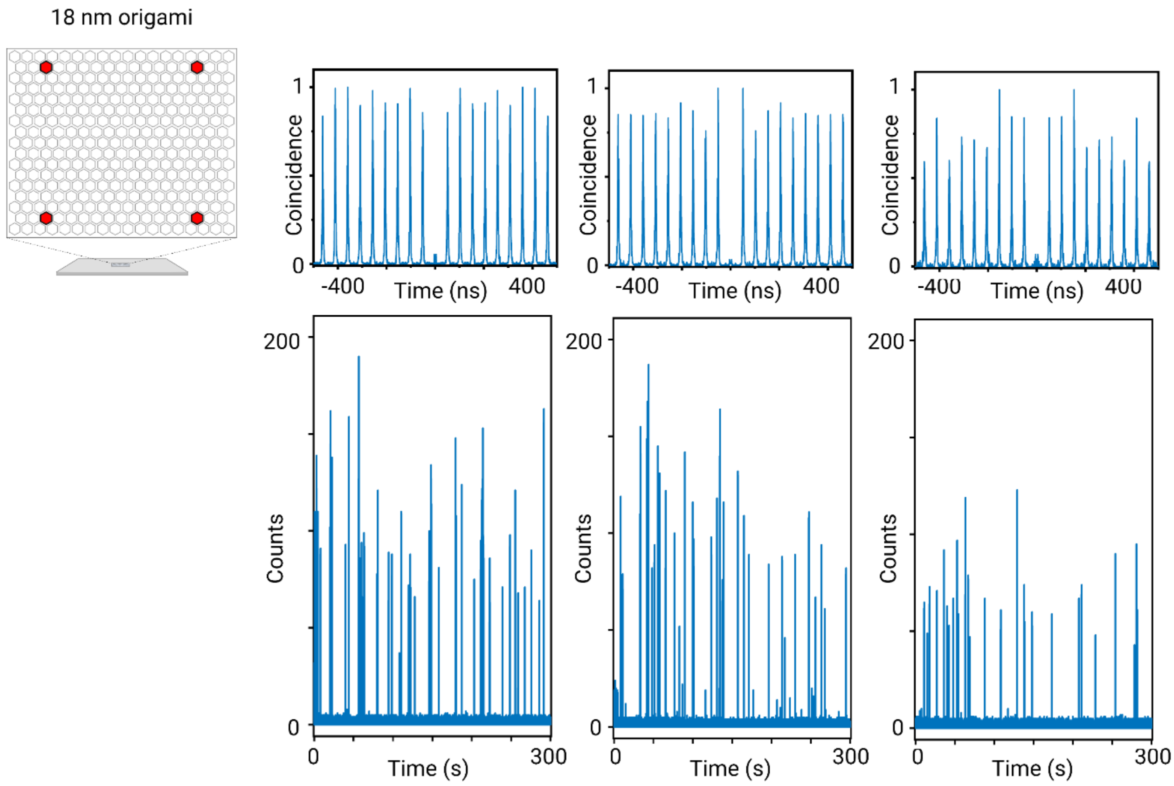

Supplementary Fig. 5. Fluorescence trajectories and corresponding normalized interphoton time (coincidence) histogram measured for the entire trajectory of 18 nm DNA origami measured by single-molecule sensitive confocal fluorescence microscopy in photoswitching buffer excited at 640 nm with 2.5 kW cm^{-2} (1 ms binning).

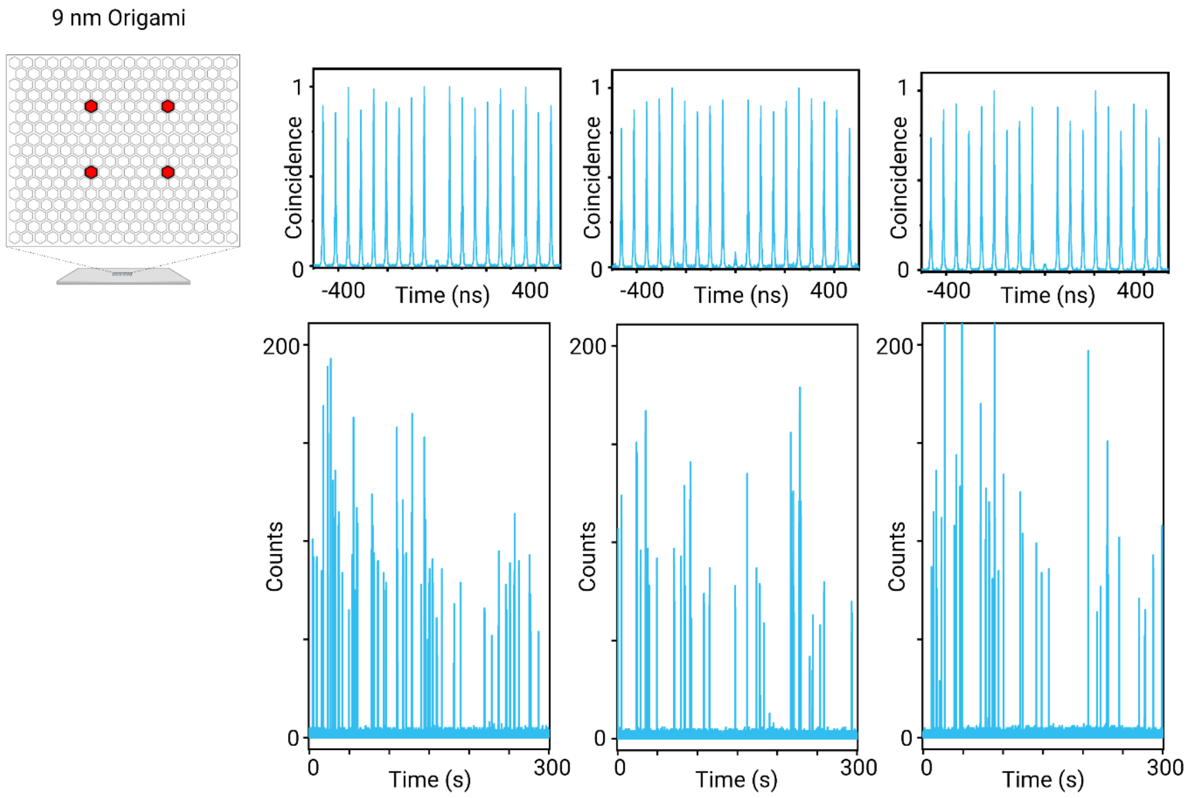

Supplementary Fig. 6. Fluorescence trajectories and corresponding normalized interphoton time (coincidence) histogram measured for the entire trajectory of 9 nm DNA origami measured by single-molecule sensitive confocal fluorescence microscopy in photoswitching buffer excited at 640 nm with 2.5 kW cm^{-2} (1 ms binning).

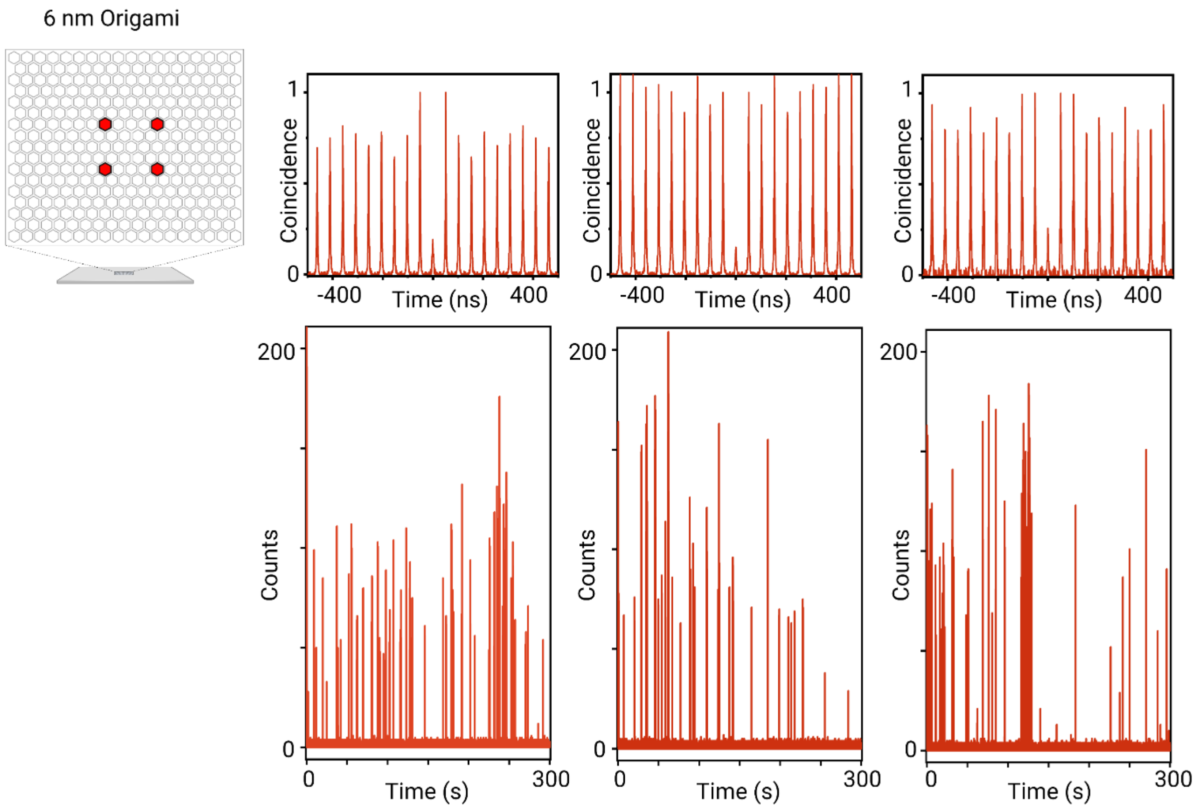

Supplementary Fig. 7. Fluorescence trajectories and corresponding normalized interphoton time (coincidence) histogram measured for the entire trajectory of 6 nm DNA origami measured by single-molecule sensitive confocal fluorescence microscopy in photoswitching buffer excited at 640 nm with 2.5 kW cm^{-2} (1 ms binning).

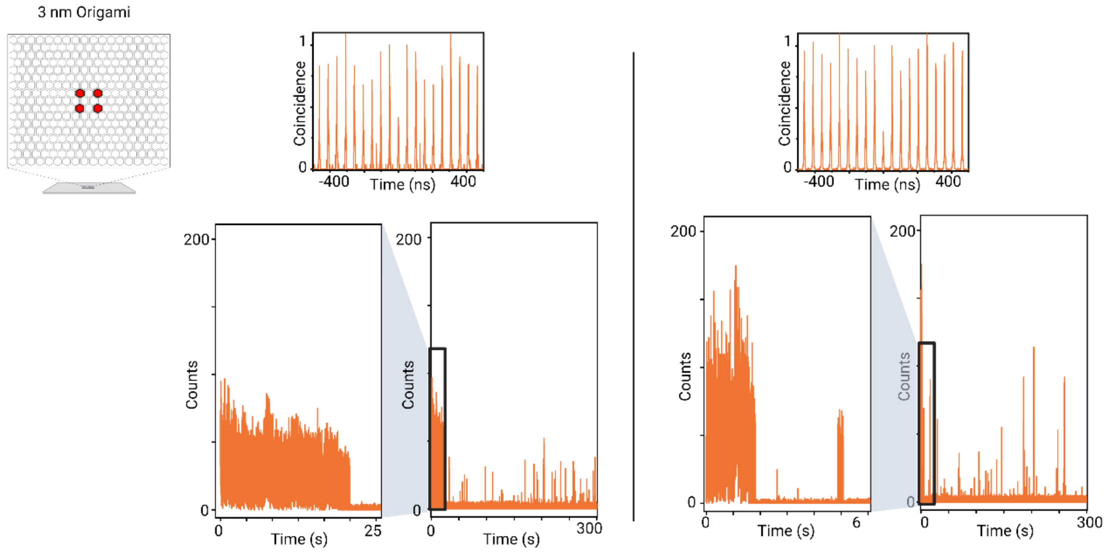

Supplementary Fig. 8. Fluorescence trajectories and corresponding normalized interphoton time (coincidence) histogram measured for the entire trajectory of 3 nm DNA origami measured by single-molecule sensitive confocal fluorescence microscopy in photoswitching buffer excited at 640 nm with 2.5 kW cm^{-2} (1 ms binning).

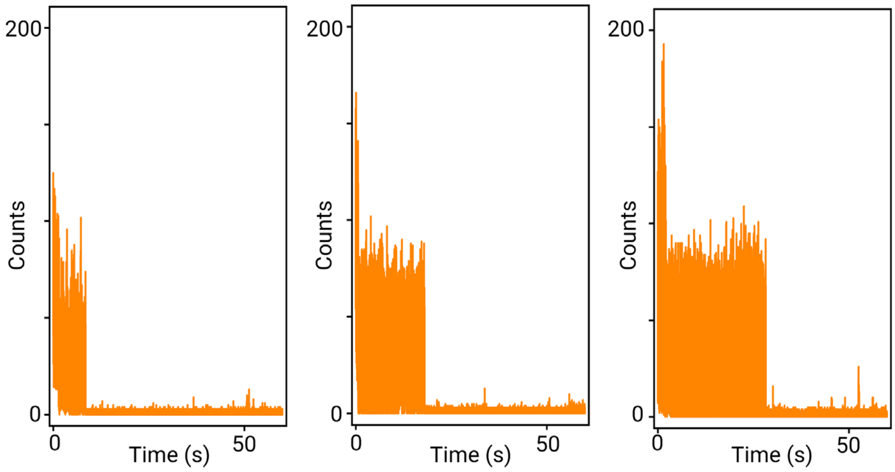

Supplementary Fig. 9. Fluorescence trajectories of 3 nm DNA origami in PBS, pH 7.6 containing 1 mM trolox/troloxquinone and an oxygen scavenging system measured by single-molecule sensitive confocal fluorescence microscopy excited at 640 nm with 2.5 kW cm^{-2} (1 ms binning).

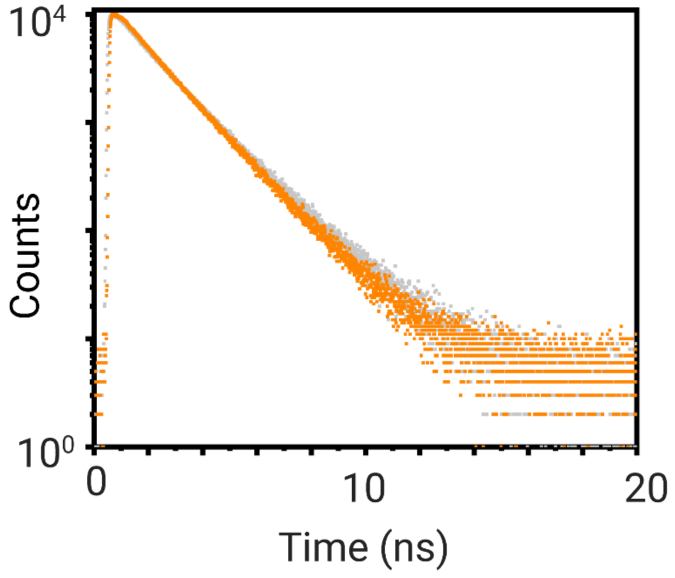

Supplementary Fig. 10. Ensemble fluorescence decays of double-stranded oligonucleotides labeled with one (5´-3´: TACGATTCGATTACGTTACCATTAGCATTGCATTAGCTTATAT-*Cy5*) and four (5´-3´: TACGATTCGATT-*Cy5*ACGTTACCATT-*Cy5*AGCATTGCATT-*Cy5*AGCTTATAT-*Cy5*) dyes measured by TCSP in PBS, pH 7.6.

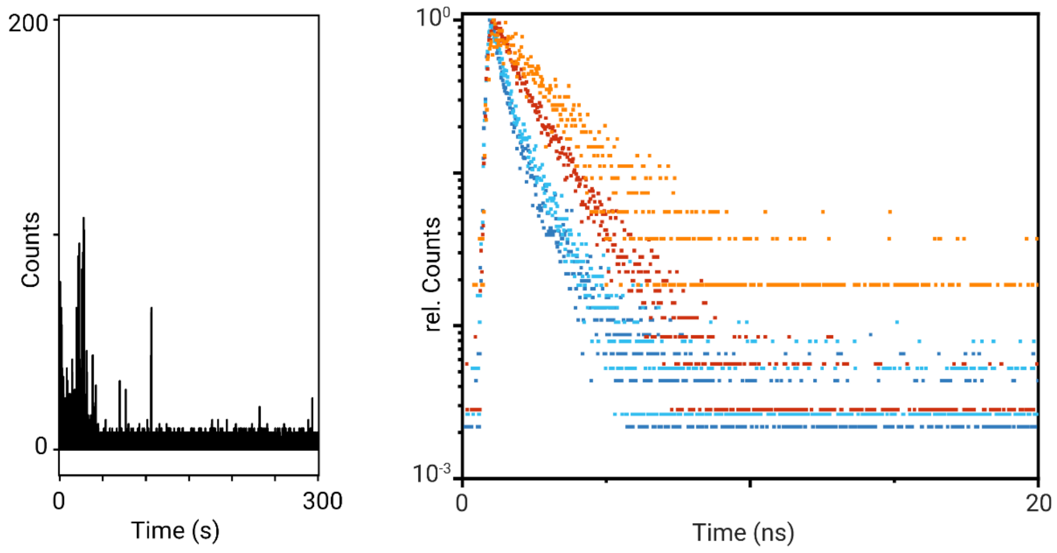

Supplementary Fig. 11. Fluorescence trajectory of a 3 nm DNA origami in photoswitching buffer and corresponding fluorescence decays recorded at different times indicate that the lifetime increases with time due to stepwise photobleaching of fluorophores and corresponding lower energy transfer efficiency (dark blue decay: 0-2 s; light blue decay: 2-25 s; red decay: 25-28 s; orange decay: 25-100 s).

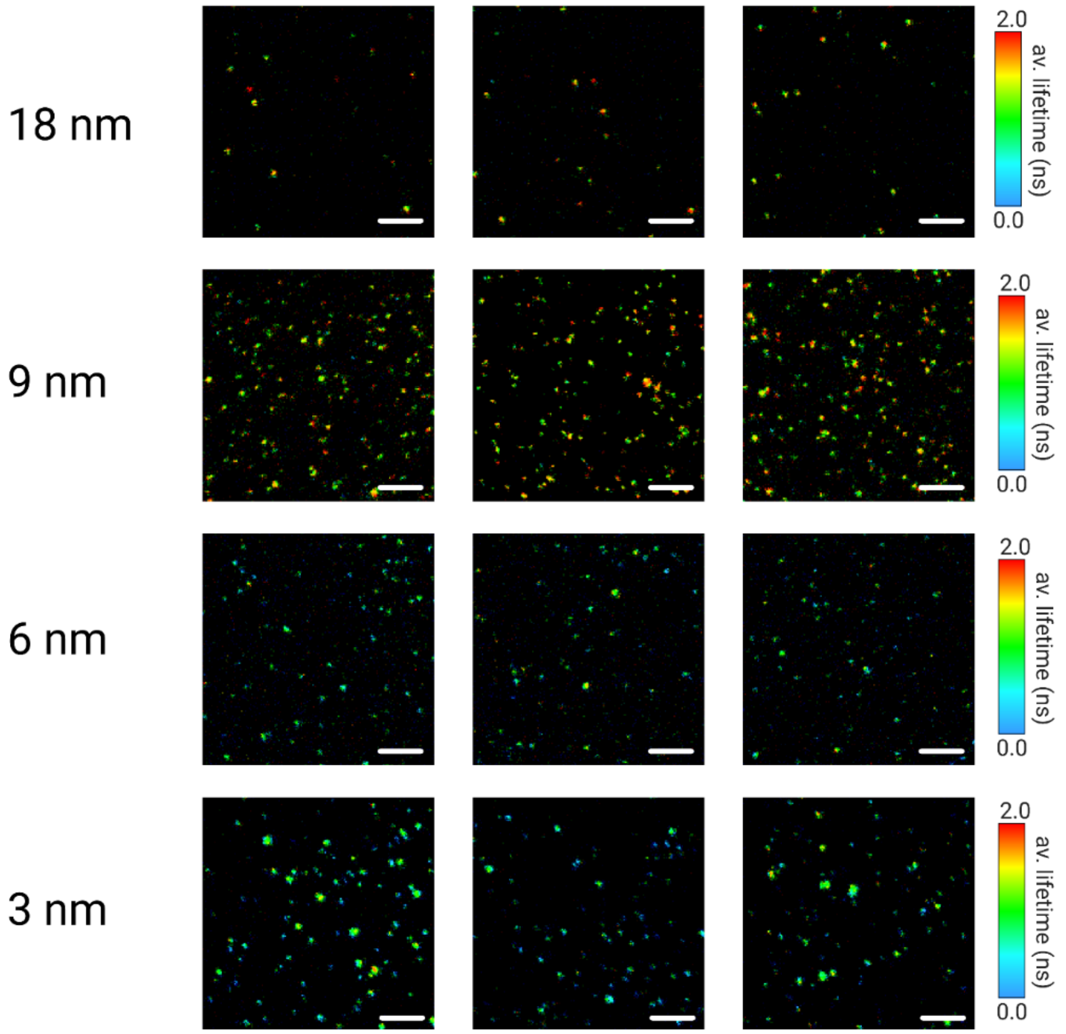

Supplementary Fig. 12. FLIM images of 18, 9, 6, 3 nm DNA origami measured in PBS, pH 7.6 containing 1 mM trolox/troloxquinone and an oxygen scavenging system measured by single-molecule sensitive confocal fluorescence microscopy excited at 640 nm with 2.5 kW cm^{-2} at an integration time of 5 µs $pixel^{-1}$. The samples were measured 5 - 10 times independently. Scale bars, 5 µm.

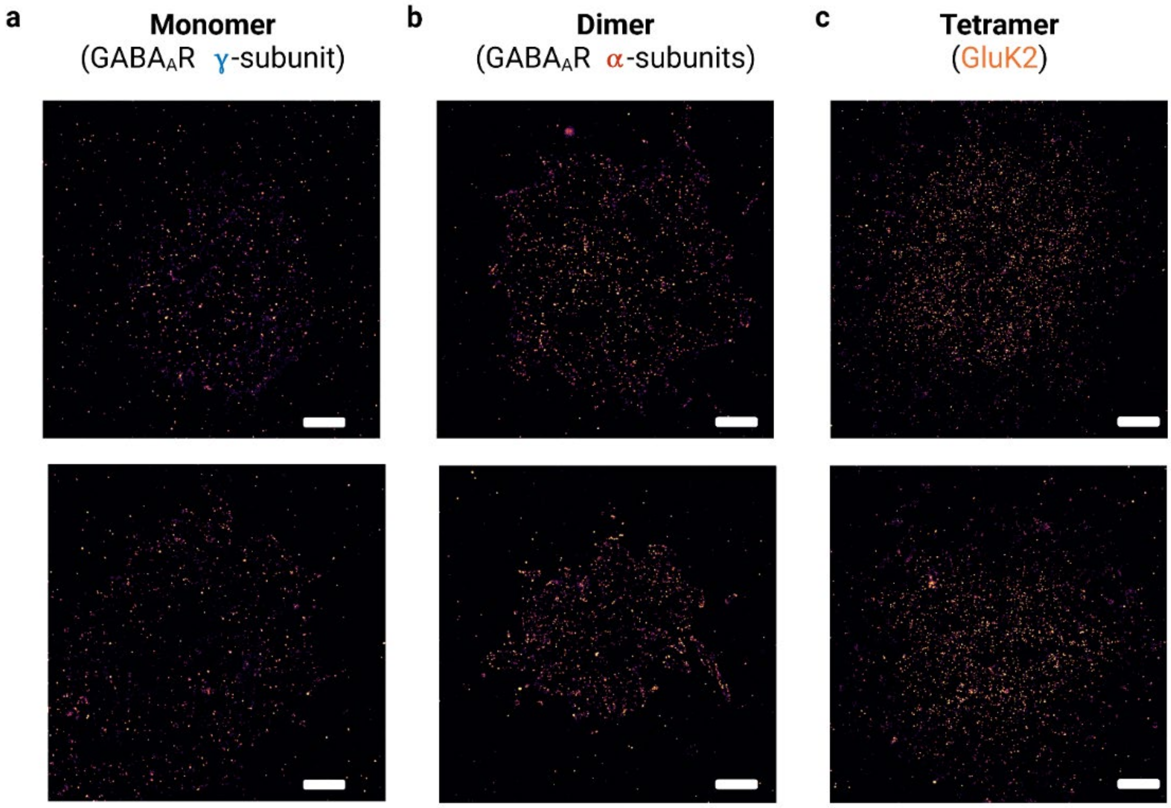

Supplementary Fig. 13. *d*STORM images of membrane receptors labeled by GCE and bioorthogonal click chemistry with Cy5 (20 nm pixel^{-1}). The samples were measured 3 - 5 times independently. Scale bars, 2 μm.

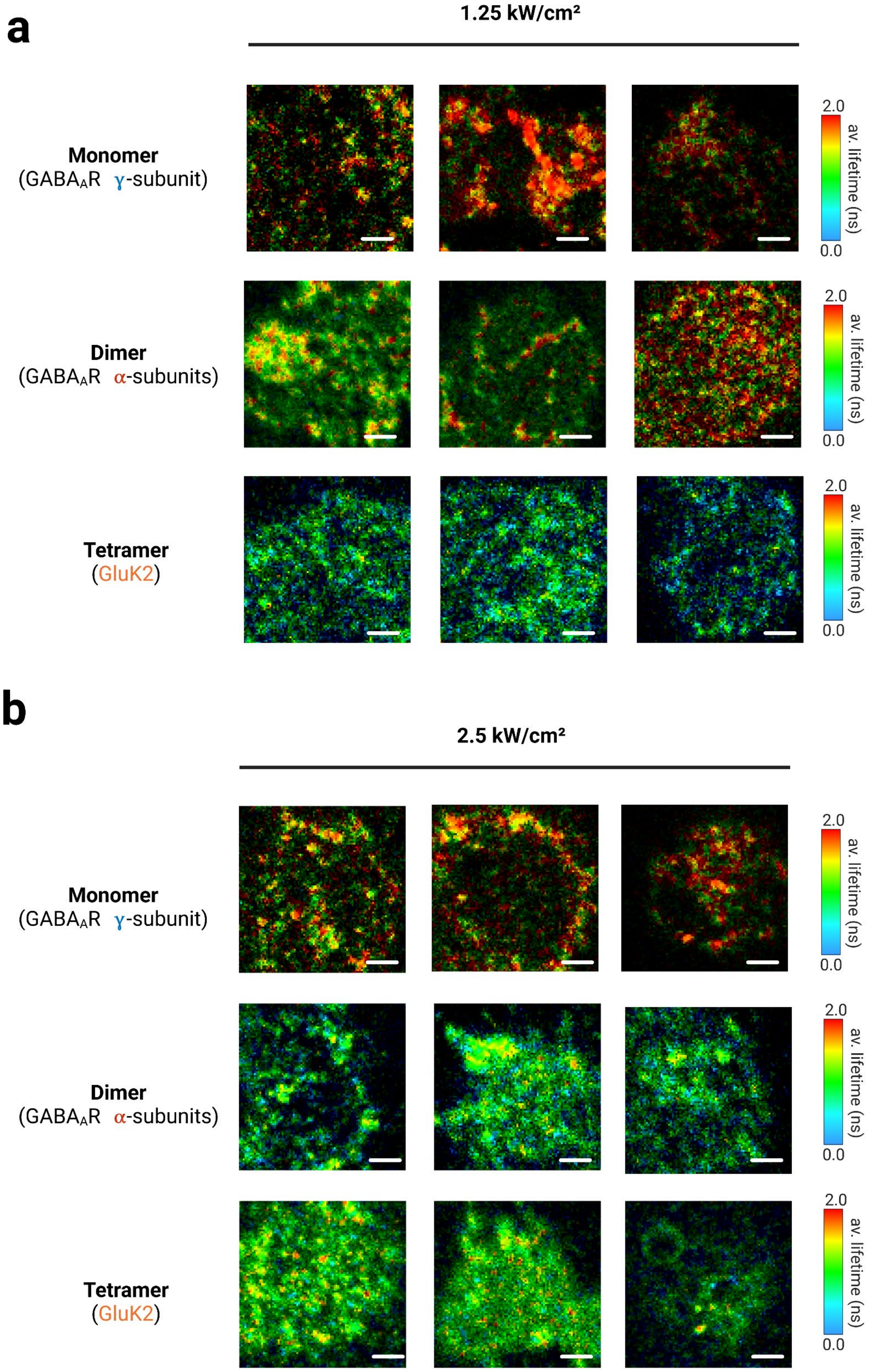

Supplementary Fig. 14. FLIM images of HEK293T cells expressing monomeric γ2-subunit of GABA-A, dimeric α2-subunit of GABA-A, and homotetrameric GluK2 receptors click-labeled with Met-Tet-Cy5 measured by confocal TCSPC imaging in photoswitching buffer at different irradiation intensity and an integration time of 5 μs $pixel^{-1}$ without applying an intensity threshold. The samples were measured 5 - 10 times independently. Scale bars, 2 μm.

Supplementary Movies

Supplementary Movies 1-5. DNA-PAINT movies (30 min) of DNA origami labeled with one (reference) or four docking strands separated by different distances recorded at a temporal resolution of 100 ms per frame.

Supplementary Movie 1. DNA-PAINT of reference DNA origamis, Scale bar, 2 µm.

Supplementary Movie 2. DNA-PAINT of 18 nm DNA origamis, Scale bar, 2 µm.

Supplementary Movie 3. DNA-PAINT of 9 nm DNA origamis, Scale bar, 2 µm.

Supplementary Movie 4. DNA-PAINT of 6 nm DNA origamis, Scale bar, 2 µm.

Supplementary Movie 5. DNA-PAINT of 3 nm DNA origamis, Scale bar,

Supplementary Movies 6-11. *d*STORM movies (10 min) of DNA origami labeled with one (reference) or four Cy5 dyes with different interfluorophore distance recorded at a temporal resolution of 5 ms per frame.

Supplementary Movie 6. *d*STORM of reference DNA origamis, Scale bar, 2 µm.

Supplementary Movie 7. *d*STORM of 18 nm DNA origamis, Scale bar, 2 µm.

Supplementary Movie 8. *d*STORM of 9 nm DNA origamis, Scale bar, 2 µm.

Supplementary Movie 9. *d*STORM of 6 nm DNA origamis, Scale bar, 2 µm.

Supplementary Movie 10. *d*STORM of 3 nm DNA origamis, Scale bar, 2 µm.

Supplementary Movie 11. *d*STORM of 3 nm DNA origamis, Scale bar, 2 µm.
